# Microbial Inoculant and Polyacrylamide Jointly Improve Cotton Root-Zone Function Under Alternating Brackish–Freshwater Irrigation

**DOI:** 10.3390/plants15152300

**Published:** 2026-07-27

**Authors:** Yilin Guo, Xiangzhuo Yu, Xingkun Wang, Hongbang Liang, Xiaoguo Mu, Guorong Ma, Jihong Zhang, Zhenhua Wang

**Affiliations:** 1 College of Water Conservancy & Architectural Engineering, Shihezi University, Shihezi 832000, China; guoyilin2026@sina.com (Y.G.); yuxiangzhuoshzu@sina.cn (X.Y.); wxk728081144@sina.com (X.W.); lianghongbang123@163.com (H.L.); muxiaoguo0104@163.com (X.M.); mgr1005@163.com (G.M.); zhangjihong_eric@163.com (J.Z.); 2 Key Laboratory of Modern Water-Saving Irrigation of Xinjiang Production & Construction Group, Shihezi University, Shihezi 832000, China; 3 Key Laboratory of Northwest Oasis Water-Saving Agriculture, Ministry of Agriculture and Rural Affairs, Shihezi 832000, China; 4 Technology Innovation Center for Agricultural Water and Fertilizer Efficiency Equipment of Xinjiang Production & Construction Corps, Shihezi 832000, China; 5 Engineering Technology Center for Comprehensive Utilization of Saline-Alkali Land of Xinjiang Production & Construction Corps, Shihezi 832000, China

**Keywords:** alternating brackish–freshwater irrigation, microbial inoculant, polyacrylamide, rhizosphere regulation, root development, nutrient uptake

## Abstract

Alternating brackish–freshwater irrigation is a promising strategy for improving the utilization of marginal water resources in arid cotton (*Gossypium hirsutum* L.) production; however, its effectiveness is often limited by salt-induced physicochemical stresses, including sodium-induced soil structural degradation, osmotic stress, and reduced rhizosphere biological activity. This study investigated whether the combined application of microbial inoculant and polyacrylamide (PAM) could enhance root-zone functioning and plant performance under alternating brackish–freshwater irrigation. A controlled greenhouse pot experiment was conducted with five treatments, including conventional irrigation (CI), alternating irrigation (AI), AI combined with microbial inoculant (AI + B), AI combined with PAM (AI + PAM), and AI combined with microbial inoculant and PAM (AI + B + PAM). Soil water–salt conditions, physical properties, nutrient availability, microbial activity, root growth, and plant nutrient uptake were determined, and partial least squares path modeling (PLS-PM) was used to evaluate soil–root–plant interactions. Alternating irrigation reduced soil salinity and sodium accumulation compared with conventional irrigation, with electrical conductivity of the 1:5 soil–water extract (EC_1:5_), Na^+^, and sodium adsorption ratio (SAR) decreasing by 14.68%, 16.21%, and 14.27%, respectively; under AI conditions, PAM increased water-stable aggregates by 22.54%, while microbial inoculant increased microbial biomass carbon by 33.47%. The combined AI + B + PAM treatment produced the greatest improvement in plant performance, increasing biomass, N uptake, P uptake, and K uptake by 28.79%, 47.37%, 48.00%, and 60.80%, respectively, compared with AI alone. PLS-PM supported a hypothesized pathway in which PAM-associated physical conditioning and microbial inoculant-mediated biochemical activation converged on root development, which was positively linked to nutrient acquisition and plant growth. These findings indicate that integrating microbial inoculant with PAM has potential to enhance root-zone resilience and cotton growth under alternating brackish–freshwater irrigation conditions, providing insights for the development of amendment strategies in saline soils. Further field validation is required before broader agricultural application.

## 1. Introduction

Freshwater scarcity has become a major constraint on sustainable agricultural production in arid and semi-arid regions [1]. This challenge is particularly acute in northwestern China, where intensive cotton (*Gossypium hirsutum* L.) cultivation depends heavily on irrigation under extremely limited freshwater availability [2,3]. In these water-deficient oasis agroecosystems, brackish groundwater is widely distributed and constitutes an important unconventional water resource with considerable development potential [4]. Compared with surface freshwater supplies, brackish groundwater is often more spatially accessible, less seasonally variable, and available in sufficient quantities to provide supplemental irrigation during critical crop growth stages [4,5]. Previous regional assessments have indicated that rational exploitation of brackish groundwater can substantially relieve pressure on high-quality freshwater resources while enhancing irrigation resilience under increasing climatic variability and water-allocation uncertainty [6,7]. Consequently, improving the efficient agricultural utilization of brackish water has become an important strategic pathway for sustaining cotton production and strengthening water-use security in saline–alkaline agricultural systems.

Alternating brackish–freshwater irrigation has been widely recognized as an effective strategy for reducing salinity risks while improving the utilization efficiency of marginal water resources. By periodically alternating saline and freshwater inputs, this strategy disrupts sustained salt accumulation and facilitates intermittent salt leaching from the root zone [8,9]. Previous studies have shown that alternating irrigation can effectively alleviate root-zone salinity stress and sustain crop growth under moderate brackish-water conditions [8,10]. However, its effectiveness is often constrained by sodium-induced clay dispersion and structural degradation, which can impair soil hydraulic conductivity and restrict root-zone functioning [11]. Irrigation scheduling alone may therefore be insufficient to fully restore favorable root-zone conditions in saline–alkaline soils, where clay dispersion and aggregate instability often require complementary soil amendment strategies [12,13,14,15,16]. These limitations highlight the need for complementary amendments that not only stabilize soil physical structure but also improve rhizosphere biological functioning. Therefore, combining physical conditioners such as PAM with biologically active amendments such as microbial inoculant may provide a more integrated strategy for root-zone regulation.

To overcome the limitations of alternating brackish–freshwater irrigation alone, complementary physical and biological soil amendments have been increasingly investigated. Polyacrylamide (PAM), in particular, has been widely applied to stabilize soil structure, enhance aggregate formation, and improve soil water retention under saline conditions [17,18]. By reinforcing aggregate stability and reducing clay dispersion, PAM improves pore connectivity and hydraulic conductivity, thereby mitigating the negative effects of sodium accumulation on root-zone functioning. In addition to physical amendments, microbial inoculants containing beneficial microorganisms have been shown to improve nutrient availability and stimulate microbial activity in the rhizosphere. These microorganisms can promote mineral nutrient solubilization through organic acid production, enzyme secretion, and enhanced rhizosphere biochemical turnover, thereby facilitating the release of nutrients from slowly available soil pools [19,20,21]. Together, PAM and microbial inoculants can operate in a complementary manner, with PAM providing a more favorable physical habitat and microbial inoculant enhancing biochemical mobilization, potentially leading to stronger plant responses than either amendment alone. Despite growing evidence for the benefits of individual amendments, few studies have systematically evaluated their integrated effects under alternating brackish–freshwater irrigation, particularly with respect to root-zone regulation and subsequent plant performance. Therefore, the combined application of PAM and microbial inoculant is hypothesized to provide complementary physical and biochemical support, addressing the limitations of irrigation alone and creating a more favorable environment for cotton growth in saline–alkaline soils.

In this study, we address these gaps using cotton seedlings grown under alternating brackish–freshwater irrigation as a model system, with PAM and microbial inoculant applied individually or in combination under saline–alkaline conditions. However, the combined use of PAM and indigenous salt-tolerant potassium-solubilizing microbial inoculant under alternating brackish–freshwater irrigation, as well as the associated soil–root–plant response pathway, has not been systematically tested. We further hypothesized that their combined application would improve root development and nutrient acquisition mainly through complementary physical and biological regulation, rather than necessarily through a significant statistical interaction. To test these hypotheses, we focused on three linked objectives: (i) to determine whether PAM mainly regulates root-zone physical conditions; (ii) to assess whether microbial inoculant mainly enhances rhizosphere biochemical activity; and (iii) to examine whether these complementary responses are associated with improved root development, nutrient uptake, and plant growth performance. By linking these responses, we aim to clarify how coordinated physical and biochemical regulation improves cotton performance under alternating brackish–freshwater irrigation and to provide a mechanistic basis for sustainable brackish-water utilization in saline–alkaline cotton production systems.

## 2. Materials and Methods

### 2.1. Experimental Soil and Irrigation Water 

The pot experiment was conducted during the cotton growing season in 2025, lasting approximately four months, in a controlled greenhouse at the Water-saving Irrigation Experimental Station of Shihezi University, Shihezi, Xinjiang, China (44°18′ N, 86°03′ E). Experimental soil was collected from the 0–20 cm plough layer of a long-term mulched drip-irrigated cotton field located in the 121st Regiment of the Xinjiang Production and Construction Corps. The soil was classified as gray desert soil according to the Chinese Soil Taxonomy, corresponding approximately to Calcaric Cambisols in the World Reference Base for Soil Resources (WRB) classification. After collection, soil samples were air-dried at room temperature, gently crushed, and passed through a 2-mm sieve to remove visible plant residues and gravel before use. 

The experimental soil was characterized by moderate salinity and weak alkalinity, representing a typical saline–alkaline cotton production soil in arid oasis regions of Xinjiang. The initial soil (0–20 cm) had a bulk density of 1.41 g cm^−3^, which was determined using three undisturbed soil cores following the core method. The initial soil also had a pH of 8.07, electrical conductivity (EC_1:5_) of 0.93 dS m^−1^, organic matter content of 7.10 g kg^−1^, available nitrogen of 65.01 mg kg^−1^, available phosphorus of 14.61 mg kg^−1^, and available potassium of 196.27 mg kg^−1^.

Freshwater was obtained from the irrigation supply system of the experimental station and had an EC_1:5_ of approximately 0.65 dS m^−1^. A NaCl-based simulated brackish water was prepared by dissolving analytical-grade NaCl in freshwater to achieve an EC_1:5_ of 3.0 dS m^−1^. In addition, irrigation water with an EC_1:5_ of 1.83 dS m^−1^ was prepared by mixing freshwater and brackish water at a 1:1 volume ratio and was used for the conventional irrigation treatment.

### 2.2. Experimental Design

A completely randomized pot experiment was conducted with five treatments: conventional brackish-water irrigation without amendment (CI), alternating brackish–freshwater irrigation without amendment (AI), alternating irrigation with microbial inoculant (AI + B), alternating irrigation with PAM (AI + PAM), and alternating irrigation with combined microbial inoculant and PAM (AI + B + PAM). Each treatment was replicated four times, resulting in a total of 20 pots. Plastic pots (25 cm diameter, 30 cm height) were filled with 13 kg of air-dried soil and planted with three cotton seedlings (Supplementary Figure S1). Basal fertilization was uniformly applied before transplanting by thoroughly mixing 1.31 g urea, 0.46 g KH_2_PO_4_, and 0.16 g K_2_SO_4_ into the soil of each pot, providing 0.60 g N, 0.105 g P, and 0.20 g K pot^−1^, equivalent to 0.24 g P_2_O_5_ and 0.24 g K_2_O pot^−1^. To avoid nutrient limitation during growth, supplementary fertigation was applied uniformly to all treatments twice during the experimental period, with the same application timing, nutrient concentration, and solution volume for each pot. Consequently, the total nutrient input throughout the experiment reached 1.20 g N, 0.48 g P_2_O_5_, and 0.48 g K_2_O pot^−1^.

Seedlings were raised with freshwater before transplantation. After seedling establishment, irrigation treatments were initiated. The CI treatment received 500 mL of irrigation water with an EC of 1.83 dS m^−1^ during each irrigation event, corresponding to the average salinity level of the alternating irrigation treatment. The AI-related treatments received 250 mL of brackish water (EC = 3.0 dS m^−1^) followed by 250 mL of freshwater (EC = 0.65 dS m^−1^) during each irrigation event. Irrigation was applied whenever soil volumetric water content declined below 15%, with 500 mL of water supplied per pot during each irrigation event. A total of ten irrigation events were applied throughout the experiment. Pots were arranged in a completely randomized design within the greenhouse and repositioned weekly to minimize spatial variation in environmental conditions.

### 2.3. Application of Microbial Inoculant and PAM

The microbial inoculant used in this study was a composite microbial inoculant (M1) developed by our research group. Strain A10 was isolated from saline–alkaline cotton fields in Xinjiang and identified as an indigenous salt-tolerant plant growth-promoting rhizobacterium. The inoculant consisted of *Pseudomonas aeruginosa* A10 and *Bacillus mucilaginosus* S1 mixed at a 1:1 ratio based on viable cell counts [22]. Strain A10 was deposited in the China General Microbiological Culture Collection Center under the accession number CGMCC No. 32694, and its 16S rRNA gene sequence was deposited in GenBank under the accession number PP516541. Preliminary characterization confirmed that A10, S1, and M1 possessed potassium-solubilizing capacity and salt–alkaline adaptability, supporting their use as candidate plant growth-promoting inoculants under saline–alkaline conditions (Supplementary Figures S2–S4). A10 and S1 were therefore selected as the bacterial components of M1 based on their complementary potassium-solubilizing and stress-adaptive characteristics. Both strains were cultured separately in Luria–Bertani liquid medium at 30 °C and 150 rpm for 24 h, centrifuged, and resuspended in sterile deionized water to obtain a final concentration of 1 × 10^8^ CFU mL^−1^, which was selected based on our previous experimental results and related studies on potassium-solubilizing rhizobacteria application [22]. Equal volumes of the two standardized suspensions were mixed immediately before application to prepare the M1 inoculant. All procedures involving A10 were conducted under controlled laboratory and greenhouse conditions following institutional biosafety handling procedures for opportunistic bacterial strains. The A10-containing inoculant was used as an experimental material in the present pot study, and its broader agricultural application should be supported by strain-level biosafety evaluation, microbial persistence assessment, environmental risk assessment, and compliance with relevant microbial fertilizer regulations.

Microbial inoculant was applied through root-zone fertigation to ensure direct contact between the inoculum and the rhizosphere soil. The first application was conducted 10 days after seedling transplantation, and subsequent applications were performed at 7-day intervals throughout the experimental period. For each application, 5 mL of bacterial suspension was delivered to each plant using a drip irrigation cone inserted into the rhizosphere zone to simulate localized fertigation under mulched drip irrigation conditions.

The PAM used in this study was an anionic polyacrylamide with a molecular weight of 8–12 × 10^6^ Da and a hydrolysis degree of 18–22%. PAM was dissolved in deionized water to prepare a 0.1% (*w*/*v*) stock solution and thoroughly mixed into the soil before transplanting to ensure uniform distribution within the root zone. For treatments receiving PAM, the application rate was equivalent to 0.1% of dry soil weight, which was determined based on our preliminary experiments and previous studies on saline–sodic soil amendment [23].

For the combined treatment (AI + B + PAM), PAM was incorporated into the soil prior to transplantation, followed by periodic microbial inoculant application according to the same schedule as the microbial inoculant-only treatment. This treatment strategy was designed to evaluate the coordinated effects of structural conditioning by PAM and rhizosphere biochemical activation by microbial inoculant under alternating brackish–freshwater irrigation.

### 2.4. Cotton Cultivation and Management

Cotton cultivar Xinluzao 84 was used in this experiment. Seeds were surface-sterilized with 1% sodium hypochlorite solution for 10 min, thoroughly rinsed with deionized water, and germinated in seedling trays filled with sterilized vermiculite. Uniform seedlings at the two-true-leaf stage were selected and transplanted into experimental pots.

The experiment was conducted in a controlled greenhouse under an average air temperature of 28.5 ± 1.5 °C, relative humidity of 70 ± 5%, and a 16 h light/8 h dark photoperiod. Artificial LED lighting was provided at an average intensity of 18,000 lux, corresponding to an estimated photosynthetic photon flux density (PPFD) of approximately 240 μmol m^−2^ s^−1^ at canopy height. Soil volumetric water content was continuously monitored using integrated soil moisture–electrical conductivity sensors (Hengyu, China). Plants were harvested at the bolling stage, and rhizosphere soil and plant samples were collected for subsequent analyses.

### 2.5. Sample Collection and Measurements

Cotton plants and rhizosphere soil samples were collected at the bolling stage. Rhizosphere soil was collected using the root-shaking method. Briefly, plants were carefully uprooted, and loosely attached bulk soil was gently removed by shaking. Soil tightly adhering to the root surface was brushed off and collected as rhizosphere soil. Fresh soil samples were divided into two portions: one portion was stored at 4 °C for microbial biomass and enzyme activity analyses, while the remaining portion was air-dried at room temperature, ground, and passed through a 2-mm sieve for the determination of soil water–salt properties, structural characteristics, and nutrient availability.

Plant samples were separated into roots and shoots immediately after harvest. Root samples were washed thoroughly with deionized water and used for root morphological analysis. All plant samples were then oven-dried at 105 °C for 30 min, followed by drying at 75 °C to constant weight for biomass determination and nutrient analysis.

#### 2.5.1. Soil Water–Salt Indicators

Soil water content, pH, EC_1:5_, soluble Na^+^, Ca^2+^, Mg^2+^, and Sodium adsorption ratio (SAR) were determined to evaluate rhizosphere water–salt conditions. Soil water content was measured gravimetrically by oven-drying fresh soil at 105 °C for 24 h.

Soil pH and EC_1:5_ were determined in a 1:5 soil-to-water suspension using a digital pH meter (PHS-3C, Shanghai INESA, Shanghai, China) and conductivity meter (DDS-307A, Shanghai INESA, Shanghai, China), respectively. Soluble Na^+^ and K^+^ concentrations were measured using a flame photometer (FP6450, Shanghai Instrument Co., Shanghai, China), while Ca^2+^ and Mg^2+^ were determined by EDTA titration.

SAR was calculated according to the following equation:

(1) $$SAR=\frac{Na^{+}}{\sqrt{(Ca^{2+}+Mg^{2+})/2}}$$

where Na^+^, Ca^2+^, and Mg^2+^ are expressed in mmol L^−1^.

#### 2.5.2. Soil Structural and Water-Retention Properties

Soil bulk density (BD), water-stable aggregates (WSAs), mean weight diameter (MWD), and field capacity (FC) were determined to characterize soil structural and water-retention properties.

Bulk density was measured using the core method with a 100 cm^3^ stainless steel ring. Water-stable aggregate distribution was determined by wet sieving using sieves of 2.0, 1.0, 0.5, and 0.25 mm. The proportion of aggregates >0.25 mm was defined as water-stable aggregates [24]. Mean weight diameter was calculated as:

(2) $$MWD=\sum_{i=1}^{n}X_{i}W_{i}$$

where $X_{i}$ is the mean diameter of each size fraction and $W_{i}$ is the corresponding mass proportion.

Field capacity was determined using the saturation-drainage method, in which saturated soil samples were allowed to drain freely for 48 h before moisture content determination.

#### 2.5.3. Soil Nutrient Availability and Plant Nutrient Uptake

Soil available nitrogen (AN), available phosphorus (AP), and available potassium (AK) were determined following standard analytical procedures. Available nitrogen was measured using the alkaline hydrolysis diffusion method. Available phosphorus was extracted with 0.5 mol L^−1^ NaHCO_3_ and determined using the molybdenum blue colorimetric method. Available potassium was extracted with 1 mol L^−1^ ammonium acetate and quantified by flame photometry [25].

Plant samples were digested using the H_2_SO_4_–H_2_O_2_ wet digestion method. Total nitrogen was determined using the Kjeldahl method, phosphorus by molybdenum blue spectrophotometry, and potassium by flame photometry.

Plant nutrient uptake was calculated as:

(3) Nutrientuptake =Drybiomass× Nutrientconcentration 

Root morphology, including root length, root surface area, and root volume, was measured using a root scanner (Epson Expression 12000XL, Suwa, Japan) coupled with WinRHIZO software (version WinRHIZO 5.0, Regent Instruments, Québec City, QC, Canada).

#### 2.5.4. Microbial Biomass and Soil Enzyme Activities

Soil microbial biomass carbon (MBC) was determined using the chloroform fumigation–extraction method [26]. Soil urease and alkaline phosphatase activities were measured to assess rhizosphere biological activity. Urease activity was determined using the sodium phenol–sodium hypochlorite colorimetric method based on NH_4_^+^ released after urea hydrolysis [27]. Alkaline phosphatase activity was determined using p-nitrophenyl phosphate as the substrate, and the released p-nitrophenol was quantified colorimetrically [28]. Urease activity was expressed as μg NH_4_^+^-N g^−1^ dry soil h^−1^, and alkaline phosphatase activity was expressed as μg p-nitrophenol g^−1^ dry soil h^−1^.

### 2.6. Data Analysis

All statistical analyses were conducted using R 4.3.2 [29]. For plant-level measurements, values from the three seedlings within each pot were averaged before statistical analysis, and the pot-level mean was used as one biological replicate. Data normality and homogeneity of variance were assessed using the Shapiro–Wilk and Levene’s tests, respectively, prior to statistical analysis. A two-way analysis of variance (ANOVA) was performed to evaluate the main effects of microbial inoculant (B), PAM, and their interaction (B × PAM) on soil physicochemical properties, microbial activity, root morphological traits, and plant nutrient uptake. When significant differences were detected, mean comparisons among treatments were conducted using Tukey’s honestly significant difference (HSD) test at the 0.05 probability level. Detailed two-way ANOVA results, including F-values and *p*-values for the main effects of microbial inoculant, PAM, and their interaction, are provided in Supplementary Table S3. The raw mean ± SD values for all measured variables across treatments are provided in Supplementary Table S4.

To visualize the overall treatment responses across multiple indicators, all measured variables were standardized using Z-score normalization and displayed as a clustered heatmap. Hierarchical clustering was performed based on Euclidean distance using complete linkage. Partial least squares path modeling (PLS-PM) was applied to quantify the relationships among soil physical regulation, rhizosphere biochemical activation, root development, and plant performance under alternating brackish–freshwater irrigation [30]. Given the limited sample size, the final PLS-PM was simplified by excluding highly correlated indicators to reduce model complexity. Representative variables were then retained based on their biological relevance and their ability to characterize the core function of each latent construct. The latent variable “Physical regulation” included soil bulk density, water-stable aggregates, mean weight diameter, and field capacity; “Biochemical activation” included available nutrients, microbial biomass carbon, and enzyme activities; “Root development” included root biomass, root length, and root surface area; and “Plant performance” included biomass and nutrient uptake. Model goodness-of-fit and path coefficients were used to evaluate direct and indirect effects among latent variables. All figures were generated using the ggplot2, pheatmap, and patchwork packages, while PLS-PM analysis was conducted using the plspm package. Statistical significance was defined at *p* < 0.05.

## 3. Results

### 3.1. Effects of Different Treatments on Root-Zone Water–Salt Conditions

**Figure 1:**
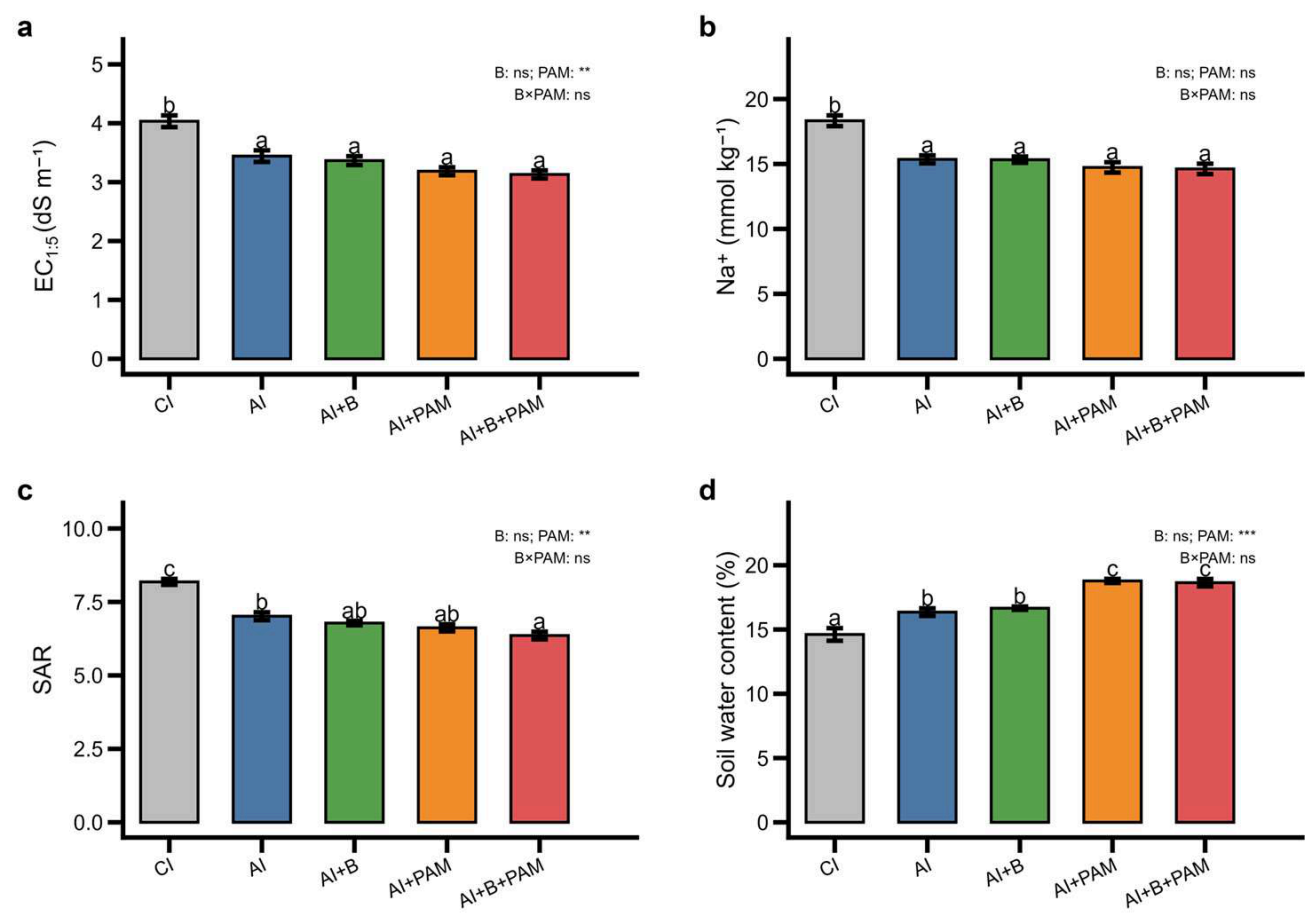
Effects of alternating saline–freshwater irrigation and amendment application on soil salinity characteristics and water content. Note: Effects of PAM and microbial inoculant on soil electrical conductivity (EC_1:5_), (**a**), Na^+^ concentration (**b**), sodium adsorption ratio (SAR), (**c**), and soil water content (SWC), (**d**) under alternating brackish–freshwater. CI, conventional irrigation; AI, alternating brackish–freshwater; B, microbial inoculant; PAM, polyacrylamide. Bars represent mean ± SD. Different lowercase letters indicate significant differences among treatments at *p* < 0.05. The two-way ANOVA was performed using the AI-based factorial treatments only, namely AI, AI + B, AI + PAM, and AI + B + PAM. Significance of two-way ANOVA is shown in each panel: ns, not significant; **, *p* < 0.01; ***, *p* < 0.001.

As shown in Figure 1, compared with conventional irrigation (CI), alternating irrigation with brackish and freshwater (AI) significantly improved the soil water–salt environment. Under AI, soil EC_1:5_, Na^+^ concentration, and SAR decreased from 4.034 to 3.442 dS m^−1^, 18.327 to 15.356 mmol kg^−1^, and 8.183 to 7.015, corresponding to reductions of 14.68%, 16.21%, and 14.27%, respectively. In contrast, soil water content increased from 14.614% to 16.351%, representing an 11.89% increase. These results indicate that alternating brackish–freshwater irrigation effectively alleviated soil salinity and sodium accumulation while improving soil water retention.

Based on AI, amendment application further optimized root-zone water–salt conditions. PAM-containing treatments produced the strongest responses, with AI + PAM and AI + B + PAM achieving the lowest salinity indicators and highest soil water content. In particular, AI + B + PAM reduced SAR to 6.358, while AI + PAM and AI + B + PAM increased soil water content to 18.778% and 18.643%, respectively. Two-way ANOVA showed that PAM significantly affected EC, SAR, and soil water content, whereas neither microbial inoculant nor the B × PAM interaction significantly affected these variables. Overall, alternating brackish–freshwater irrigation served as the primary measure for mitigating salt and sodium stress, while PAM was the dominant factor further enhancing soil water retention and reducing sodicity risk.

### 3.2. Effects of Different Treatments on Soil Structure and Water-Holding Capacity

**Figure 2:**
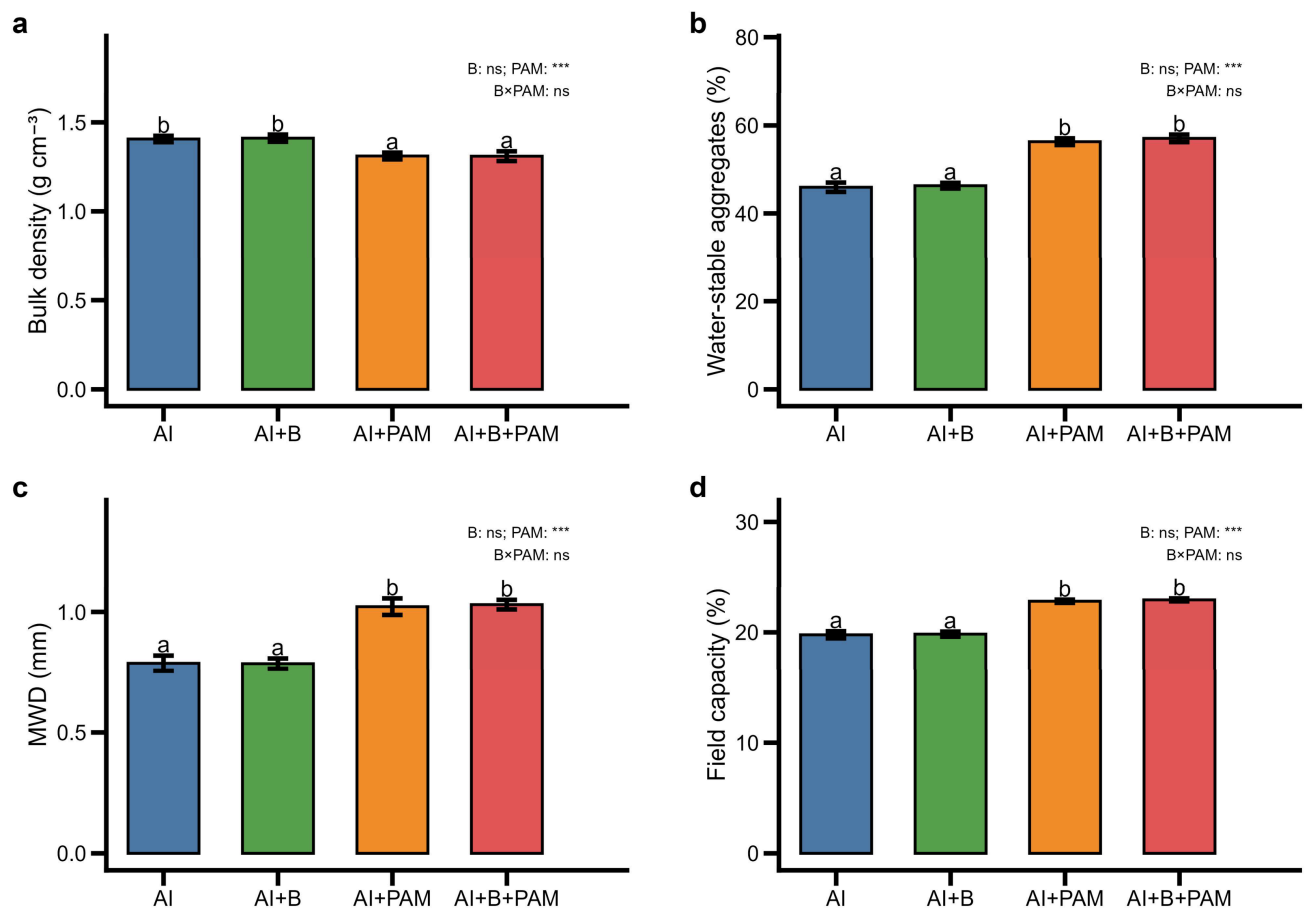
Effects of microbial inoculant and PAM on soil physical properties under alternating brackish–freshwater irrigation. Note: Effects of microbial inoculant and PAM on soil physical properties under alternating brackish–freshwater irrigation: (**a**) bulk density (BD), (**b**) water-stable aggregates (WSA), (**c**) mean weight diameter (MWD), and (**d**) field capacity (FC). AI, alternating brackish–freshwater irrigation without amendment; B, microbial inoculant; PAM, polyacrylamide; B + PAM, combined application of microbial inoculant and PAM. Different lowercase letters indicate significant differences among treatments at *p* < 0.05. The two-way ANOVA was performed using the AI-based factorial treatments only, namely AI, AI + B, AI + PAM, and AI + B + PAM. Significance of two-way ANOVA is shown in each panel: ns, not significant; ***, *p* < 0.001.

As shown in Figure 2, PAM markedly improved soil physical structure, whereas microbial inoculant alone had little effect. Compared with AI, PAM reduced bulk density from 1.407 to 1.311 g cm^−3^ and increased water-stable aggregates, MWD, and field capacity from 45.948% to 56.303%, 0.787 to 1.022 mm, and 19.778% to 22.818%, corresponding to changes of −6.82%, 22.54%, 29.86%, and 15.37%, respectively. The AI + B + PAM treatment showed a similar improvement pattern, with bulk density further reduced to 1.310 g cm^−3^ and water-stable aggregates, MWD, and field capacity increased to 57.064%, 1.030 mm, and 22.942%, respectively.

Two-way ANOVA showed that PAM significantly affected bulk density, water-stable aggregates, and MWD, whereas neither microbial inoculant nor the B × PAM interaction significantly affected these variables. For field capacity, treatment differences were mainly observed between PAM-containing and non-PAM treatments. Overall, PAM was the dominant factor improving soil structure by reducing compaction and enhancing aggregate stability, aggregate size, and water-holding capacity.

### 3.3. Effects of Different Treatments on Soil Fertility and Microbial Activity

**Figure 3:**
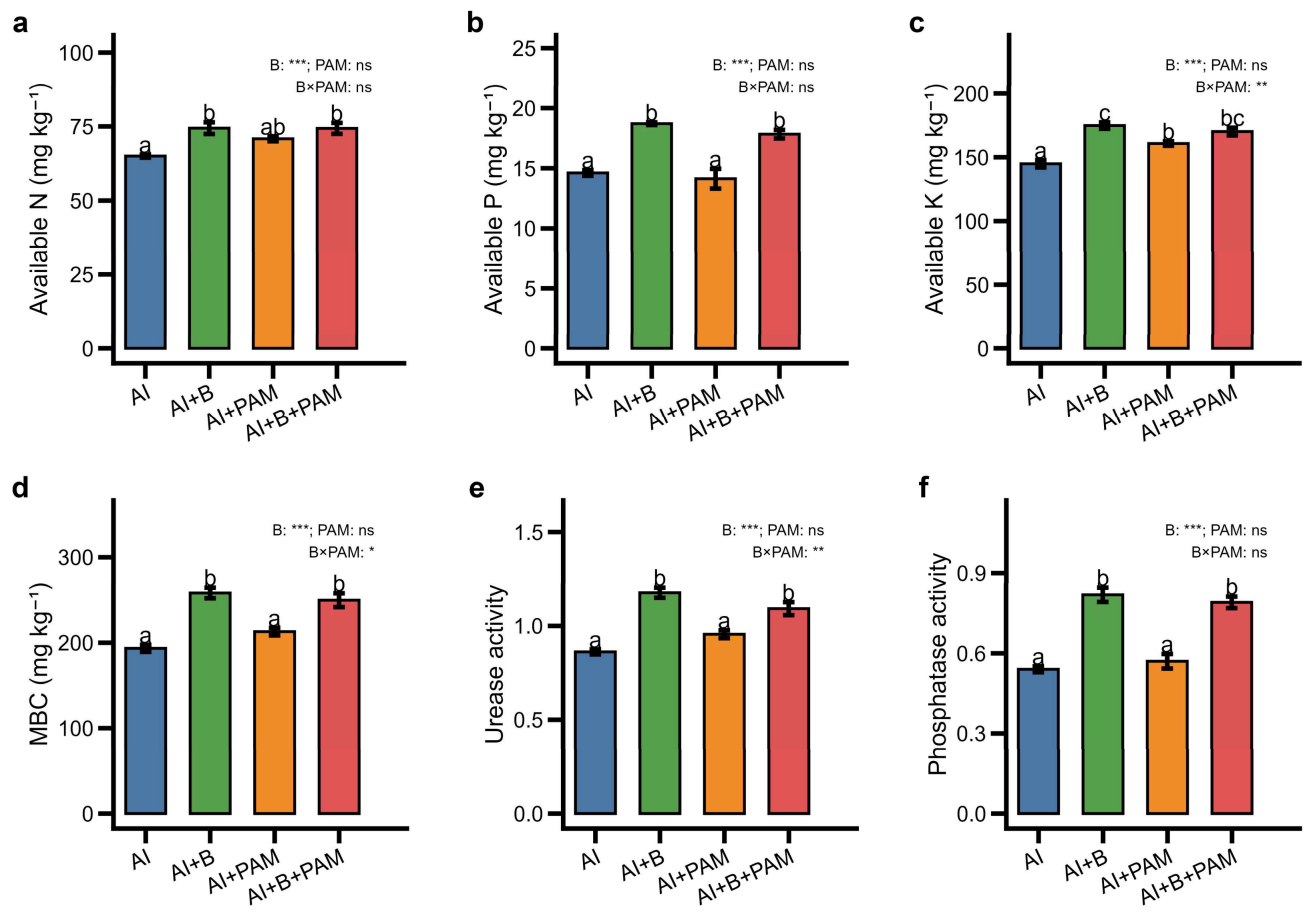
Effects of microbial inoculant and PAM on soil fertility and microbial activity under alternating brackish–freshwater irrigation. Note: (**a**) available nitrogen (AN), (**b**) available phosphorus (AP), (**c**) available potassium (AK), (**d**) microbial biomass carbon (MBC), (**e**) urease activity, and (**f**) phosphatase activity. AI, control; B, microbial inoculant; PAM, polyacrylamide. Different lowercase letters indicate significant differences among treatments (*p* < 0.05); ns, not significant. The two-way ANOVA was performed using the AI-based factorial treatments only, namely AI, AI + B, AI + PAM, and AI + B + PAM. Significance of two-way ANOVA is shown in each panel: ns, not significant; *, *p* < 0.05; **, *p* < 0.01; ***, *p* < 0.001.

As shown in Figure 3, microbial inoculant markedly improved soil nutrient availability, microbial biomass, and enzyme activities, whereas PAM alone showed limited effects. Compared with AI, microbial inoculant increased available N, available P, and available K from 65.005 to 74.422 mg kg^−1^, 14.614 to 18.705 mg kg^−1^, and 144.895 to 174.836 mg kg^−1^, respectively. Microbial biomass carbon also increased from 193.460 to 258.224 mg kg^−1^, accompanied by increases in urease and phosphatase activities from 0.862 to 1.177 and 0.540 to 0.819, respectively.

The AI + B + PAM treatment maintained the highest overall nutrient and biochemical responses, although the additional contribution of PAM beyond microbial inoculant alone was limited. Two-way ANOVA confirmed that microbial inoculant significantly affected all measured soil fertility and biological indicators, whereas PAM significantly affected only phosphatase activity. Significant B × PAM interactions were observed for available K, and microbial biomass carbon. Overall, microbial inoculant was the dominant factor enhancing nutrient availability and microbial activity under alternating brackish–freshwater irrigation.

### 3.4. Effects of Different Treatments on Cotton Root Growth

**Figure 4:**
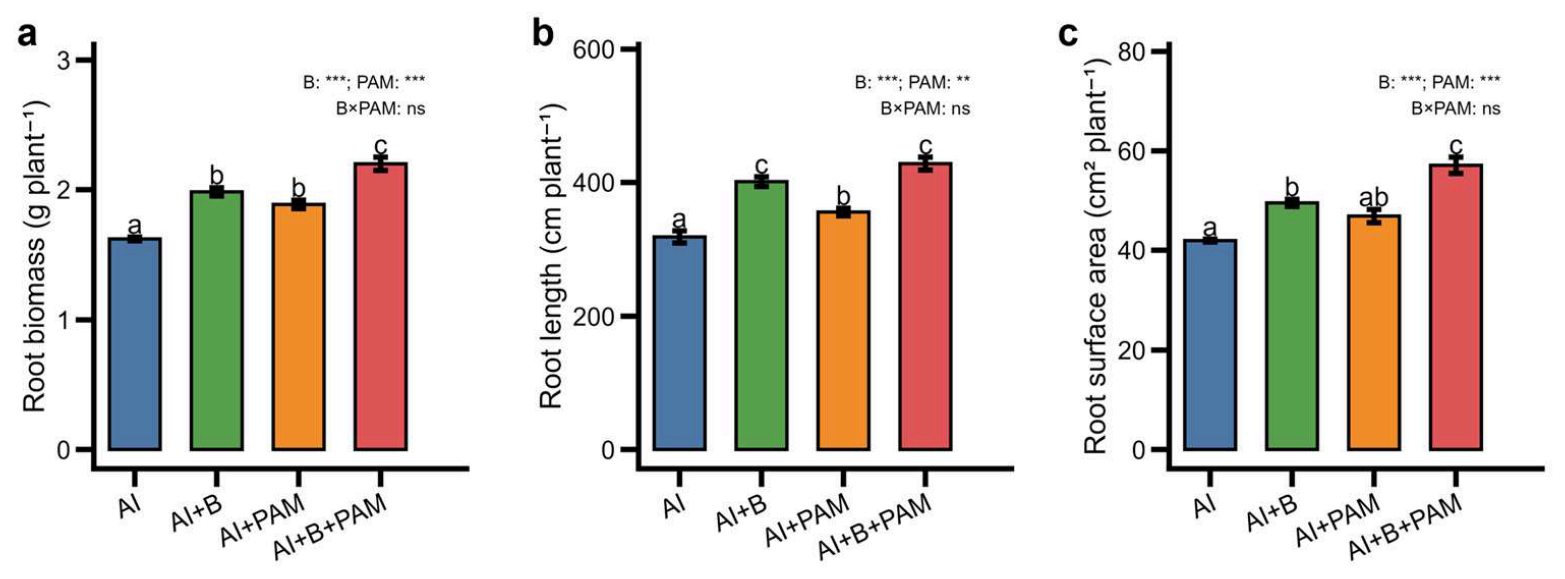
Effects of microbial inoculant and PAM on root growth under alternating brackish–freshwater irrigation. Note: (**a**) root biomass, (**b**) root length, and (**c**) root surface area. AI, control; B, microbial inoculant; PAM, polyacrylamide. Different lowercase letters indicate significant differences among treatments (*p* < 0.05); ns, not significant. The two-way ANOVA was performed using the AI-based factorial treatments only, namely AI, AI + B, AI + PAM, and AI + B + PAM. Significance of two-way ANOVA is shown in each panel: ns, not significant; **, *p* < 0.01; ***, *p* < 0.001.

Microbial inoculant and PAM both promoted root growth under alternating brackish–freshwater irrigation (Figure 4). Compared with AI, B significantly increased root biomass, root length, and root surface area by 22.41%, 26.07%, and 18.09%, respectively. PAM also increased root biomass, root length, and root surface area by 16.42%, 11.74%, and 11.77%, respectively. The AI + B + PAM treatment showed the greatest root growth. Compared with AI, AI + B + PAM significantly increased root biomass from 1.62 to 2.20 g plant^−1^, root length from 318.25 to 428.25 cm plant^−1^, and root surface area from 41.94 to 57.09 cm^2^ plant^−1^, corresponding to increases of 35.80%, 34.56%, and 36.13%, respectively. These increases were greater than those observed under B or PAM alone, indicating that the combined application produced the strongest treatment-level improvement. Two-way ANOVA showed that both microbial inoculant and PAM significantly affected root biomass, root length, and root surface area. However, the B × PAM interaction was not significant.

### 3.5. Effects of Different Treatments on Plant Growth and Nutrient Uptake

**Figure 5:**
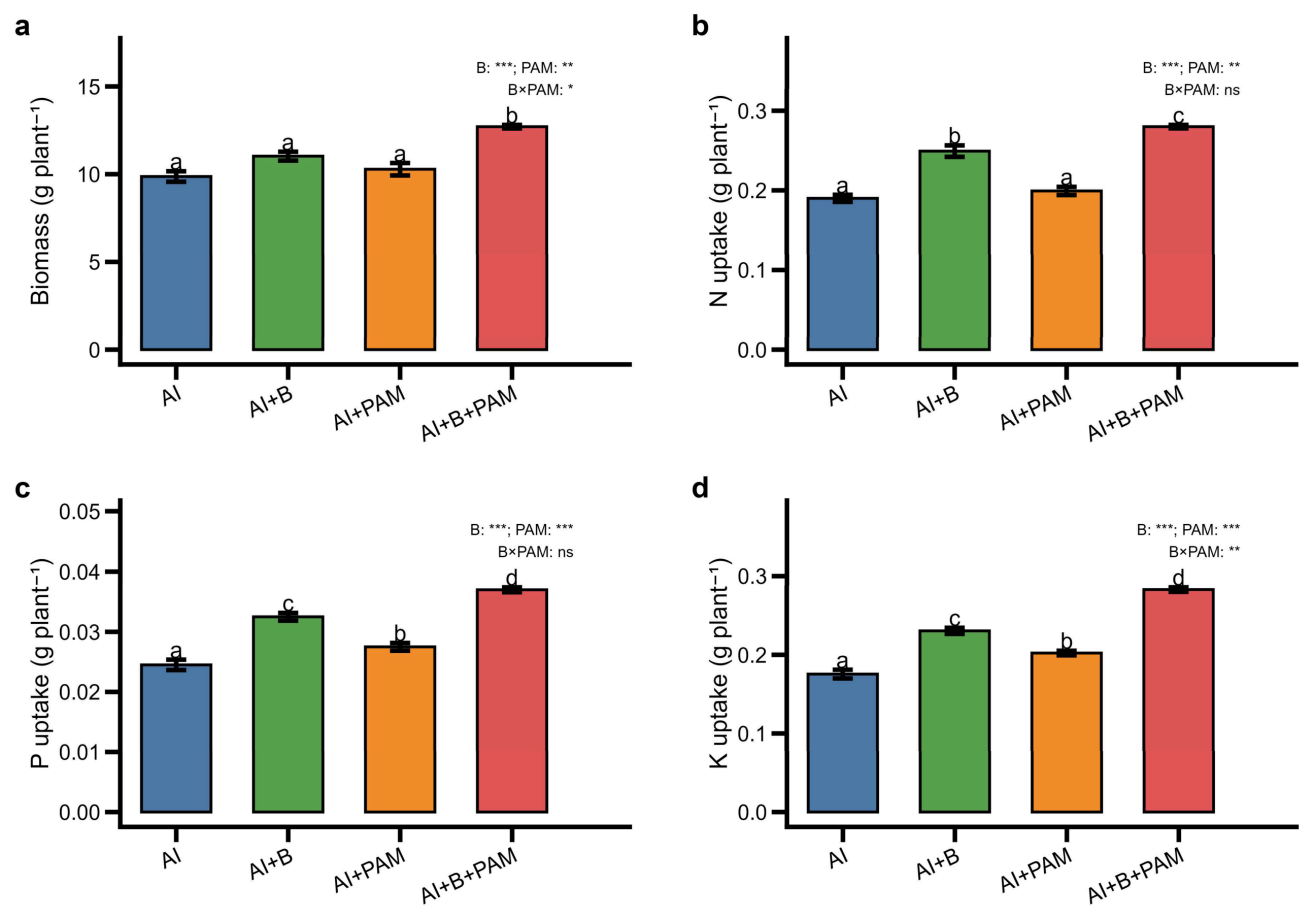
Effects of microbial inoculant and PAM on plant biomass and nutrient uptake under alternating brackish–freshwater irrigation. Note: (**a**) biomass, (**b**) N uptake, (**c**) P uptake, and (**d**) K uptake. AI, control; B, microbial inoculant; PAM, polyacrylamide. Different lowercase letters indicate significant differences among treatments (*p* < 0.05); ns, not significant. The two-way ANOVA was performed using the AI-based factorial treatments only, namely AI, AI + B, AI + PAM, and AI + B + PAM. Significance of two-way ANOVA is shown in each panel: ns, not significant; *, *p* < 0.05; **, *p* < 0.01; ***, *p* < 0.001.

As shown in Figure 5, microbial inoculant and PAM significantly affected plant biomass and nutrient uptake. For biomass, B and PAM alone increased biomass by 11.76% and 4.21% compared with AI, respectively, but these increases were not significant. In contrast, AI + B + PAM significantly increased biomass from 9.867 to 12.708 g plant^−1^, representing a 28.79% increase. This trend was consistent with the enhanced aboveground growth observed in Supplementary Figure S5, with the combined treatment producing the highest numerical biomass response.

AI + B + PAM also produced the highest nutrient uptake, increasing N uptake from 0.190 to 0.280 g plant^−1^, P uptake from 0.025 to 0.037 g plant^−1^, and K uptake from 0.176 to 0.283 g plant^−1^, corresponding to increases of 47.37%, 48.00%, and 60.80%, respectively, compared with AI. N uptake was mainly promoted by microbial inoculant, whereas both amendments independently enhanced P uptake. K uptake exhibited a significant interactive response, with the combined application producing a greater increase than either B (0.231 g plant^−1^) or PAM (0.202 g plant^−1^) alone. These results indicate that improved nutrient acquisition, particularly K uptake, contributed directly to enhanced plant growth under alternating brackish–freshwater irrigation.

### 3.6. Integrated Pathways Linking Root-Zone Regulation and Plant Performance

The standardized response heatmap showed distinct response patterns among treatments. AI + B mainly enhanced nutrient availability and microbial biochemical activity, whereas AI + PAM primarily improved water retention and soil physical conditions. The combined AI + B + PAM treatment showed the strongest overall responses in root growth, biomass accumulation, and nutrient uptake, indicating that the combined application promoted plant performance through coordinated improvements in root-zone water–fertility conditions.

**Figure 6:**
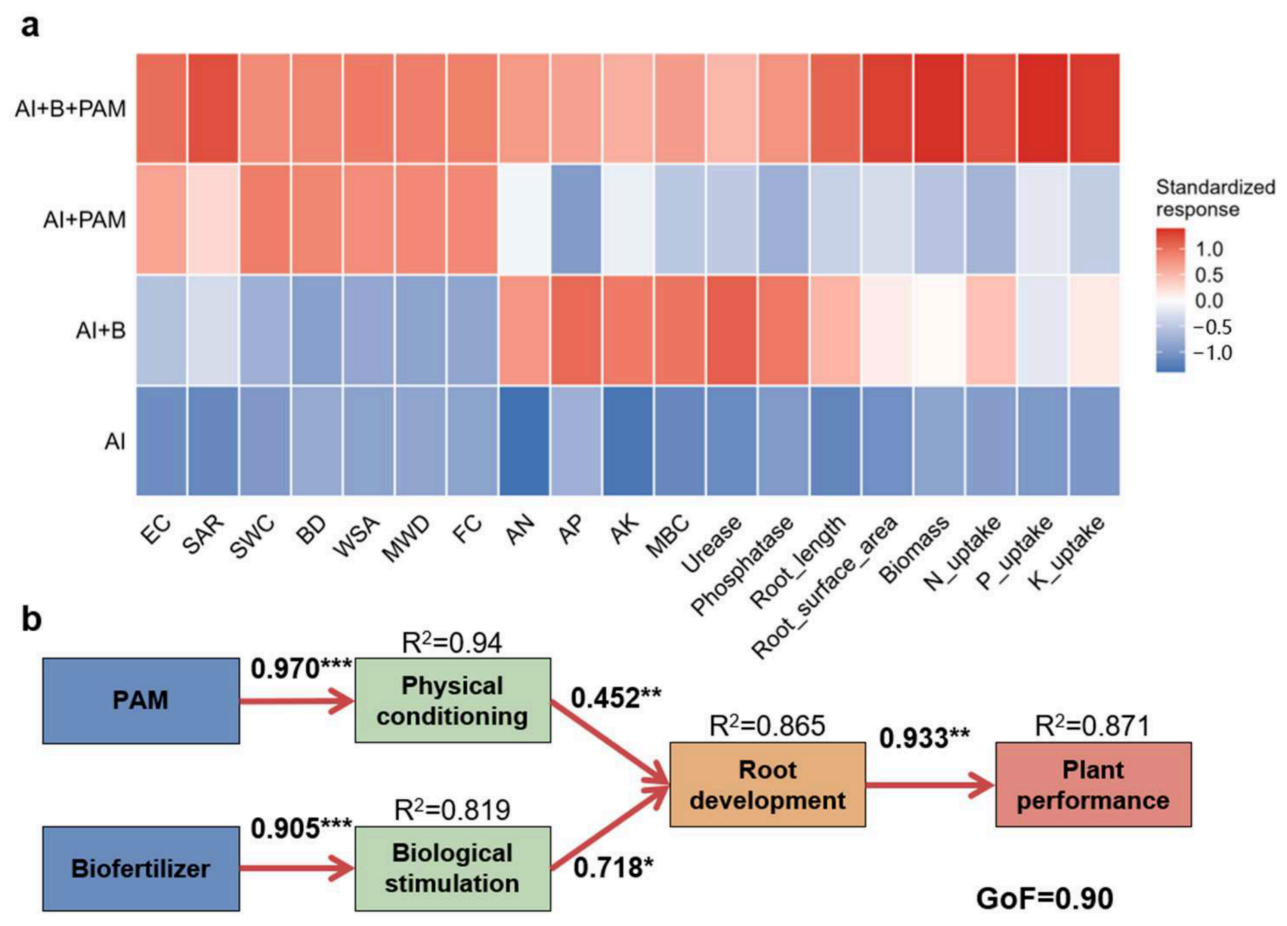
Standardized response patterns and PLS-PM pathway of microbial inoculant and PAM effects under alternating brackish–freshwater irrigation. Note: (**a**) Standardized response heatmap of root-zone water–salt status, soil physical conditions, nutrient availability, microbial biochemical activity, root growth, biomass, and nutrient uptake. AI, control under alternating brackish–freshwater irrigation; B, microbial inoculant; PAM, polyacrylamide; B + PAM, combined microbial inoculant and PAM application. EC_1:5_, SAR, and BD were reverse-transformed before standardization so that higher standardized values indicate more favorable root-zone conditions. The heatmap shows relative response patterns rather than statistical significance. (**b**) Simplified partial least squares path modeling (PLS-PM) describing the major regulatory pathway. Values on arrows indicate standardized path coefficients. R^2^ values are shown for endogenous latent variables. Physical conditioning was represented by SWC and WSA; Biological stimulation was represented by AK and MBC; Root development was represented by root biomass and root surface area; Plant performance was represented by biomass and K uptake. GoF indicates goodness-of-fit. Bootstrap validation showed that the 95% confidence intervals of all displayed paths did not include zero. Significance levels are indicated as follows: *, *p* < 0.05; **, *p* < 0.01; ***, *p* < 0.001.

The simplified PLS-PM further supported the hypothesized pathway linking root-zone regulation and plant performance (Figure 6). PAM strongly and positively affected the Physical conditioning module, with a path coefficient of 0.970, while microbial inoculant strongly promoted the Biological stimulation module, with a path coefficient of 0.905. Both Physical conditioning and Biological stimulation positively contributed to Root development, with path coefficients of 0.452 and 0.718, respectively. Root development then exerted a strong positive effect on Plant performance, with a path coefficient of 0.933. The detailed standardized path coefficients and bootstrap confidence intervals are provided in Supplementary Table S5. The model explained a high proportion of variance in Physical conditioning, Biological stimulation, Root development, and Plant performance, with R^2^ values of 0.940, 0.819, 0.865, and 0.871, respectively. The overall goodness-of-fit was 0.900. Bootstrap validation showed that the 95% confidence intervals of all major paths did not include zero, indicating that the simplified pathway was stable. These results suggest that PAM and microbial inoculant were linked to plant performance through two distinct but convergent hypothesized pathways: PAM mainly improved physical conditioning, while microbial inoculant mainly enhanced biological stimulation, and both pathways ultimately converged on root development.

## 4. Discussion

### 4.1. Root-Zone Physical Regulation Under Alternating Brackish–Freshwater Irrigation

Alternating brackish–freshwater irrigation effectively alleviated root-zone salt and sodium accumulation while improving soil moisture conditions, indicating its important role in regulating the hydraulic environment of saline–alkaline soils. This finding is consistent with previous studies showing that alternating fresh–brackish water irrigation and freshwater leaching can mitigate root-zone salinization by disrupting sustained salt accumulation and promoting salt displacement from the root zone [8,9]. The underlying mechanism is mainly associated with repeated redistribution of soil water and solutes during alternating irrigation cycles, which facilitates downward salt displacement and reduces the risk of upward salt return through capillary flow [9]. Under mulched drip irrigation, this process is particularly important because localized wetting fronts and reduced soil evaporation reshape water and salt transport pathways within the wetted root zone [31]. However, although alternating irrigation effectively regulates salt redistribution, irrigation scheduling alone may not fully offset the structural degradation induced by sodium-dominated alkaline conditions, particularly clay dispersion and aggregate destabilization [32]. Therefore, alternating brackish–freshwater irrigation should be regarded as a hydrological baseline that establishes favorable water–salt conditions for subsequent physical stabilization of the root zone.

Building upon this hydraulic regulation, PAM further reinforced root-zone physical stability and enhanced the buffering capacity of the saline soil environment. The observed reductions in sodicity indicators and concurrent improvement in soil water retention suggest that PAM played a dominant role in optimizing the physical conditions of the root zone. This agrees with the well-established role of PAM as a soil conditioner that enhances particle flocculation and aggregate stabilization through polymer-bridging mechanisms [17]. Improved aggregate stability enhances pore continuity and infiltration, thereby increasing soil water storage and reducing localized salt accumulation associated with uneven saline water redistribution [33]. This structural buffering effect is particularly important in sodicity-affected soils, where clay dispersion and pore blockage can markedly impair hydraulic conductivity and accelerate physical degradation [11]. Therefore, PAM primarily functioned as a physical regulator that strengthened the structural resilience of the root zone, creating a more stable habitat for subsequent rhizosphere biochemical activation.

### 4.2. Microbial Inoculant-Mediated Rhizosphere Activation Enhances Nutrient Acquisition

Microbial inoculant markedly enhanced soil nutrient availability, microbial biomass, and enzyme activities, indicating its dominant role in regulating rhizosphere biochemical processes. Previous studies have shown that microbial inoculants can accelerate nutrient turnover by stimulating microbial metabolism and intensifying rhizosphere nutrient cycling, thereby increasing the availability of plant-accessible nutrient pools [34,35]. Tian et al. reported that potassium-solubilizing bacteria significantly increased available K in calcareous soils by secreting low-molecular-weight organic acids that promoted the dissolution of K-bearing aluminosilicate minerals [36]. Similarly, recent studies have shown that inoculation with beneficial rhizobacteria can enhance phosphatase and urease activities, thereby accelerating nutrient mineralization and increasing the release of plant-available nutrients from relatively stable soil pools [34,37]. The increased microbial biomass observed in the present study further suggests that microbial inoculant stimulated microbial proliferation and intensified rhizosphere biochemical turnover, thereby sustaining a more active nutrient transformation environment [38]. This mechanism is particularly relevant in mineral-rich gray desert soils, where substantial potassium reserves are locked in slowly available mineral forms and require microbially mediated weathering for effective release [39]. Therefore, microbial inoculant functioned as the primary biochemical driver of rhizosphere nutrient activation, providing the nutritional basis for subsequent root development and nutrient acquisition.

The enhanced rhizosphere biochemical activity was closely associated with improved root development and greater nutrient acquisition efficiency. Increased nutrient availability expands the nutrient pool accessible to roots, thereby stimulating root proliferation and enlarging the absorptive interface between roots and soil [40]. In addition, intensified microbial activity can improve rhizosphere microenvironmental conditions by promoting osmotic adjustment, ion homeostasis, and phytohormone-mediated root development, thereby favoring root elongation and lateral root formation [41,42]. Egamberdieva et al. demonstrated that inoculation with plant growth-promoting rhizobacteria significantly enhanced root development and nutrient acquisition in saline soils by stimulating rhizosphere metabolic activity [43]. In contrast, although PAM improved the physical habitat of the root zone, its limited influence on nutrient transformation suggests that structural optimization alone may be insufficient to directly stimulate nutrient acquisition without concurrent biochemical mobilization [44]. Therefore, microbial inoculant-mediated rhizosphere activation effectively translated biochemical improvements into enhanced root nutrient acquisition, providing the biological foundation for the improved plant performance observed under combined amendment application.

### 4.3. Convergent Physical–Biological Pathways Drive Plant Performance

The combined treatment was associated with the greatest improvement in plant performance, particularly for potassium uptake. The magnitude of biomass and nutrient-uptake increases under AI + B + PAM was greater than that observed under microbial inoculant or PAM alone in this study and was broadly consistent with previous reports showing that single microbial or polymer amendments can improve crop growth under saline or structurally degraded soil conditions [45]. Improved aggregate stability and water continuity likely enhanced K^+^ transport within the rhizosphere by facilitating diffusion and maintaining continuous flow pathways [46]. Meanwhile, the microbial inoculant contained the potassium-solubilizing strain *Pseudomonas aeruginosa* A10, which can release potassium from K-bearing minerals through mineral weathering and organic acid-mediated solubilization under saline conditions [22,23]. This process is likely associated with localized rhizosphere acidification resulting from proton and organic acid release during microbial metabolism, a mechanism that has also been recognized in microbial mobilization of sparingly available mineral nutrients [47]. Together with enhanced rhizosphere biochemical activity, these processes increased the supply of plant-available K from slowly accessible mineral pools. Similar synergistic effects have been reported in saline soils, where structural soil amendments improved resource transport while microbial inoculants enhanced nutrient mobilization and plant nutrient acquisition [48,49]. Therefore, the significant increase in K uptake under the combined treatment likely resulted from the coordinated enhancement of K transport and K mobilization within the root zone.

The PLS-PM analysis further supported an integrated pathway framework linking root-zone regulation to plant performance. The model indicated two distinct yet complementary associations, with PAM primarily influencing the physical regulation pathway and microbial inoculant predominantly driving the biochemical activation pathway. At the same time, microbial inoculant-mediated mineral weathering and intensified rhizosphere biochemical activity likely promoted the release of plant-available potassium from slowly accessible mineral pools [39]. Previous studies have emphasized that sustaining plant growth under saline stress requires coordinated regulation of soil physical transport processes and rhizosphere biochemical turnover rather than reliance on either process alone [44,50]. Taken together, these findings suggest a coordinated physical–biological regulation pattern in which physical conditioning enhances resource accessibility, biochemical activation mobilizes nutrient pools, and root development serves as the central integrative node linking root-zone regulation to plant growth. This integrated regulation strategy provides a useful basis for interpreting how physical and biological root-zone processes may jointly contribute to cotton performance under saline conditions. However, the present study was conducted under greenhouse pot conditions using NaCl-based simulated brackish water; therefore, field validation with mixed-ion brackish groundwater is still required before broader application in cotton production. In addition, the use of the A10-containing inoculant should be further supported by strain-level biosafety and persistence assessments.

## 5. Conclusions

This study demonstrates that microbial inoculant and polyacrylamide function through distinct but complementary pathways to alleviate root-zone constraints under alternating brackish–freshwater irrigation. PAM primarily improved the physical environment of the rhizosphere by enhancing soil structure and water retention, whereas microbial inoculant mainly stimulated rhizosphere biochemical processes by promoting nutrient mobilization and microbial activity. Their combined application generated a coordinated improvement in root-zone function that translated into enhanced root development and plant nutrient acquisition. The exploratory path analysis was consistent with the proposed pathway in which physical conditioning and biological stimulation converged on root development, which was in turn associated with improved plant performance. These findings highlight that effective management of saline–alkaline cotton systems under marginal water use requires integrated regulation of both structural and biological root-zone processes. Under the present greenhouse pot conditions, the combined biofertilizer–PAM strategy represents a promising approach for strengthening root-zone resilience and improving the sustainability of alternating brackish–freshwater irrigation in arid agroecosystems; however, future field-scale validation is required before broader application in cotton production systems.

## Data Availability

The full pot-level raw datasets are provided in the Supplementary Materials as Tables S1 and S2. The pot-level input matrix used for the exploratory PLS-PM analysis is provided as Table S5. The R scripts used for statistical analysis, figure generation, and exploratory PLS-PM analysis are provided as Code S1. Additional statistical summaries, ANOVA results, PLS-PM path coefficients, and supplementary figures are also included in the Supplementary Materials.

## Supplementary Materials

The following supporting information can be downloaded at: https://www.mdpi.com/article/10.3390/plants15152300/s1.
