# Supplementary material for: Microbial Inoculant and Polyacrylamide Jointly Improve Cotton Root-Zone Function Under Alternating Brackish–Freshwater Irrigation
Source: Plants (Basel). 2026 Jul 27;15(15):2300. doi: 10.3390/plants15152300 (PMC13467786; doi:10.3390/plants15152300)
Supplement: Supplementary file 1 [file plants-15-02300-s001.zip › plants-4386368-supplementary.pdf]

FIGURES, TABLES AND CODE

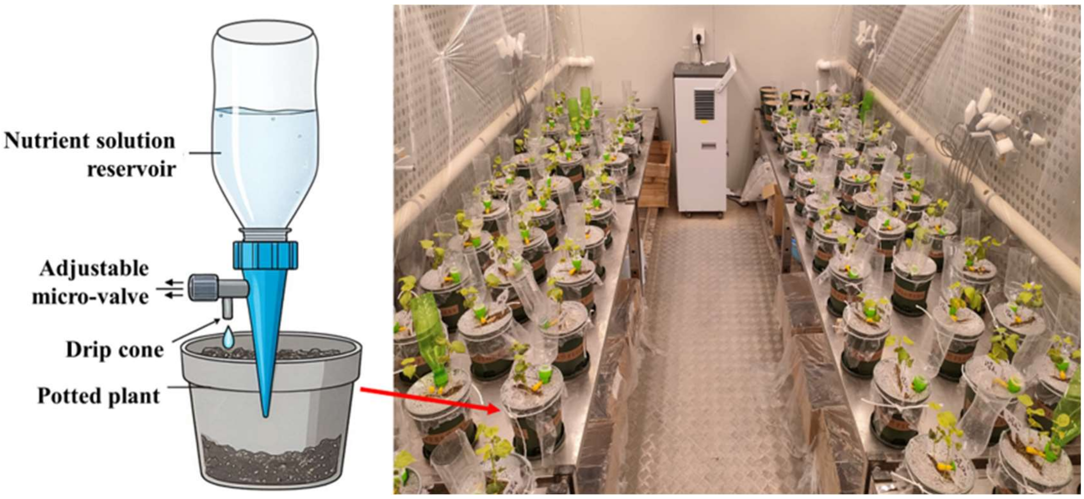

Fig. S1. Drip-fed fertigation device and experimental setup used in the pot experiment. (a) Schematic illustration of the drip-fed delivery system consisting of a nutrient solution reservoir, adjustable micro-valve, and drip cone inserted into the root zone. (b) Controlled-environment chamber showing the arrangement of cotton pots equipped with the fertigation device.

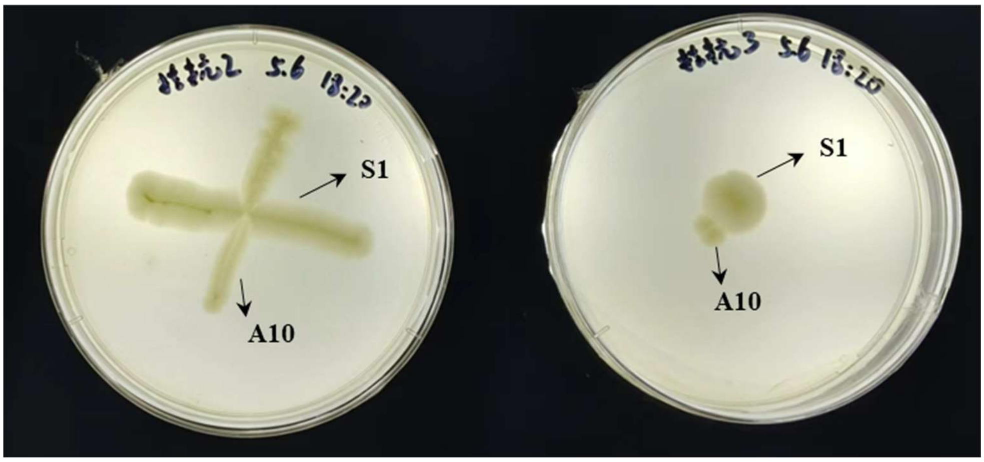

Fig. S2. Antagonism assay between strain A10 and the commercial strain S1. Dual-culture confrontation tests showing no apparent inhibitory effects between A10 and S1 on solid media. The absence of inhibition zones or growth suppression indicates that the two strains are compatible for use as a composite inoculant (M1).

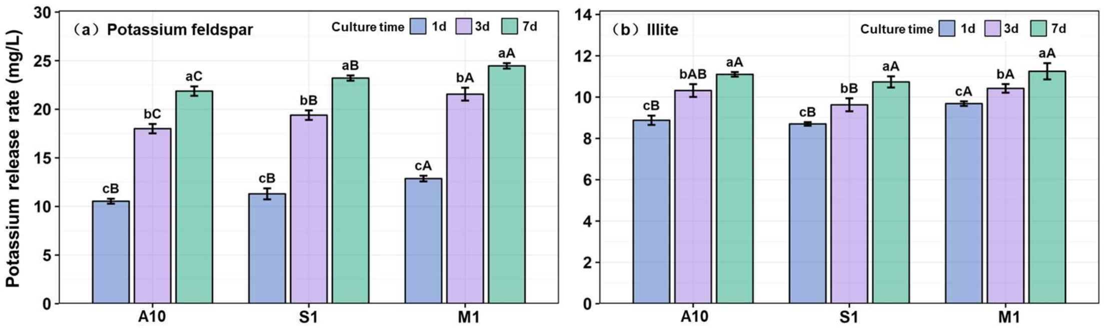

Fig. S3. Potassium-solubilizing performance of strains A10, S1, and M1 on two K-bearing minerals. (a) Potassium release from potassium feldspar and (b) illite after 1, 3, and 7 days of incubation. Bars represent mean ± SD (n = 3). Different lowercase letters indicate significant differences among culture times within the same strain, while uppercase letters denote differences among strains within the same culture time ($p < 0.05$).

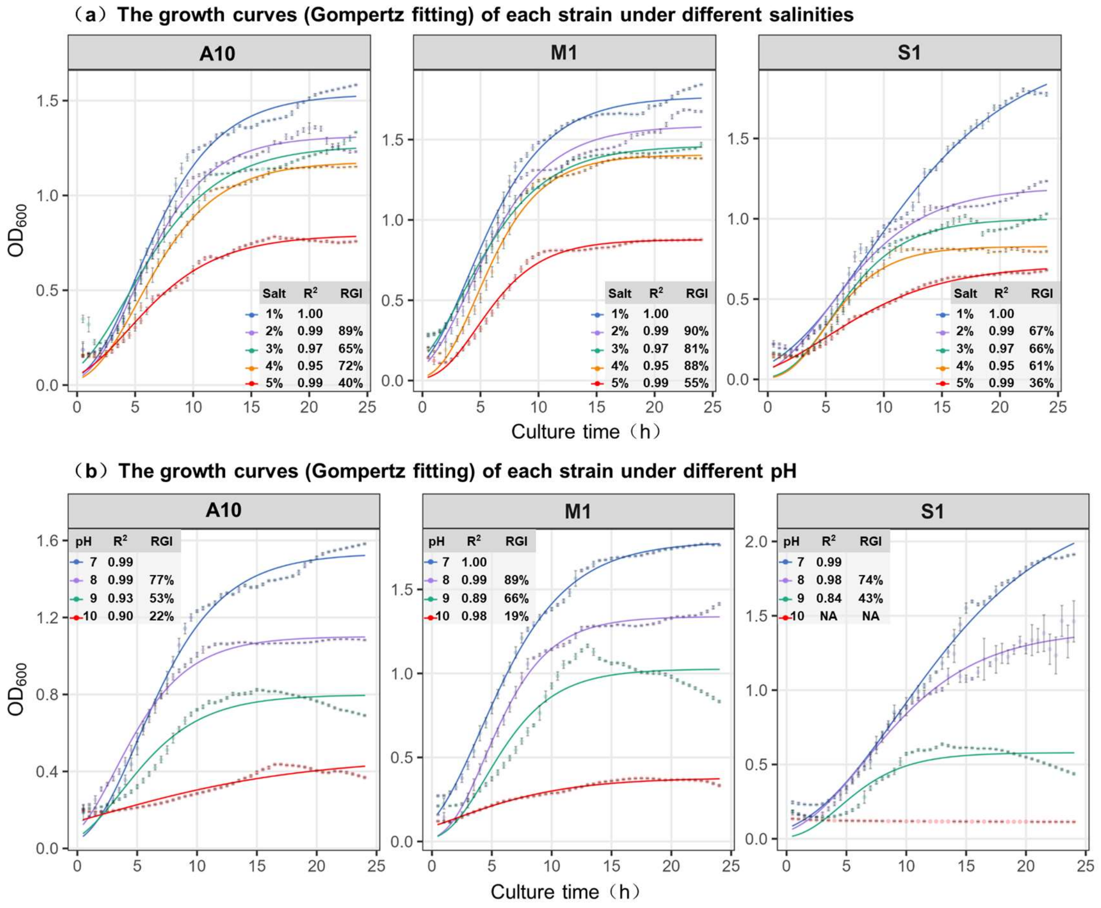

Fig. S4. Growth responses of potassium-solubilizing strains A10, M1 and S1 to salinity and pH, determined in microplate assays and fitted with the Gompertz model. Bacterial cells were revived from LB slants, pre-cultured to the logarithmic phase in LB broth, and standardized to OD_{600} = 0.05 with sterile LB. For each treatment, 200 μL of modified LB medium (1–5% NaCl for salinity assays or pH 7–10 for pH assays) was mixed with an equal volume of inoculum in sterile 96-well plates (≥3 wells per strain × treatment), sealed with gas-permeable film and incubated at 28 °C with continuous shaking in a microplate reader. OD_{600} was recorded every 30 min for 24 h and blank-corrected using uninoculated control wells. Solid lines represent mean observed OD_{600} values, whereas dashed lines show Gompertz model fits used to estimate maximum biomass (A), maximum specific growth rate (μ_{max}) and lag phase (λ). Relative growth index (RGI) was calculated from model parameters relative to the neutral control (1% NaCl, pH 7.0); inset tables present R^2 and RGI for each condition, where $RGI \geq 70\%$ with $R^2 \geq 0.95$ indicates good tolerance and $RGI \geq 85\%$ indicates strong salt or alkali tolerance.

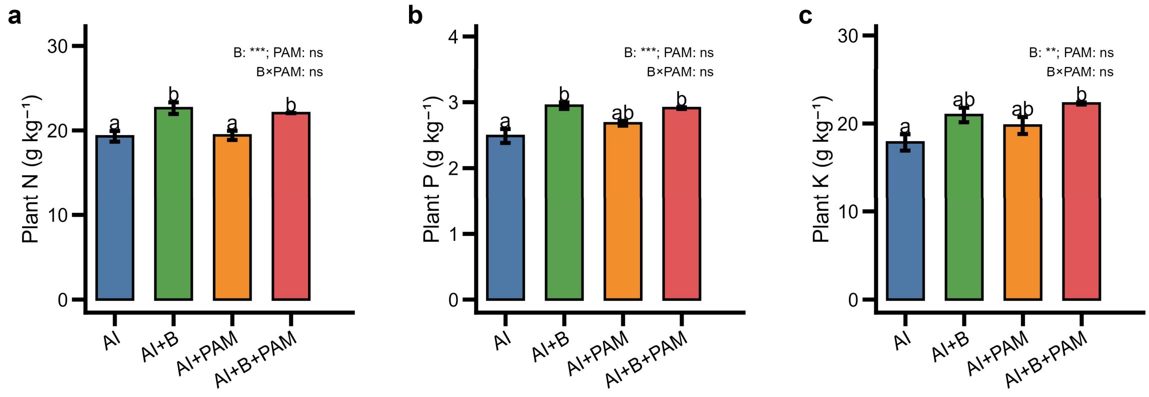

Fig. S5. Effects of microbial inoculant and PAM on plant nutrient accumulation under alternating brackish–freshwater irrigation.

Note: (a) plant N accumulation, (b) plant P accumulation, and (c) plant K accumulation. CK, control; B, microbial inoculant; PAM, polyacrylamide. Different lowercase letters indicate significant differences among treatments ($P < 0.05$); ns, not significant. Results of two-way ANOVA are shown in the upper-right corner of each panel, where Bio represents the main effect of microbial inoculant, PAM represents the main effect of polyacrylamide, and B × PAM indicates their interaction (ns, not significant; $P < 0.05$; $^*P < 0.01$; $^{**}P < 0.001$).

Table S1. Pot-level raw data for root-zone water–salt conditions, soil physical properties, and soil fertility indicators.

Treatment	Rep	Irrigation	Microbial inoculant	PAM	EC	Na	SAR	SWC	BD	WSA	MWD	FC	AN	AP	AK	MBC	Urease	Phosphatase
CI	1	Conventional	No	No	3.93	17.72	8.41	15.26	1.40	42.46	0.59	18.99	59.62	11.89	126.19	175.98	0.70	0.46
CI	2	Conventional	No	No	4.02	19.18	7.99	14.73	1.49	39.20	0.63	18.77	57.52	11.85	128.16	161.25	0.76	0.47
CI	3	Conventional	No	No	3.87	17.52	8.04	13.19	1.45	40.92	0.62	17.86	59.77	13.28	137.73	168.50	0.75	0.52
CI	4	Conventional	No	No	4.32	18.89	8.30	15.28	1.50	42.99	0.65	18.01	61.08	13.30	135.43	169.44	0.75	0.48
AI	1	Alternate	No	No	3.16	15.49	6.93	16.71	1.36	48.73	0.81	20.17	63.63	14.70	142.07	194.35	0.85	0.55
AI	2	Alternate	No	No	3.54	15.98	6.76	16.71	1.41	44.31	0.83	19.57	65.04	14.75	138.72	201.07	0.85	0.56
AI	3	Alternate	No	No	3.47	15.45	7.39	15.47	1.41	46.47	0.82	18.96	65.17	13.94	151.98	181.83	0.90	0.53
AI	4	Alternate	No	No	3.60	14.51	6.98	16.52	1.45	44.28	0.69	20.41	66.18	15.07	146.82	196.59	0.85	0.51
AI+B	1	Alternate	Yes	No	3.30	15.24	6.81	16.95	1.45	47.81	0.81	20.01	72.13	18.83	176.94	247.26	1.15	0.86
AI+B	2	Alternate	Yes	No	3.51	15.82	6.61	16.34	1.42	45.13	0.79	19.80	79.54	18.88	167.53	268.92	1.25	0.79
AI+B	3	Alternate	Yes	No	3.19	14.70	6.97	16.76	1.43	46.95	0.82	19.22	70.65	18.38	179.04	247.62	1.13	0.87
AI+B	4	Alternate	Yes	No	3.46	15.56	6.73	16.58	1.35	45.30	0.72	20.32	75.37	18.74	175.84	269.10	1.18	0.76
AI+PAM	1	Alternate	No	Yes	3.21	15.15	6.51	18.74	1.26	58.10	1.06	22.72	73.32	12.42	165.53	220.72	0.99	0.60
AI+PAM	2	Alternate	No	Yes	3.33	15.63	6.36	18.37	1.32	55.91	0.99	23.17	69.64	15.44	158.61	220.72	0.99	0.49
AI+PAM	3	Alternate	No	Yes	3.00	14.31	6.68	19.11	1.34	54.48	0.95	22.48	70.11	13.04	157.85	205.60	0.93	0.59
AI+PAM	4	Alternate	No	Yes	3.20	13.86	6.93	18.90	1.33	56.73	1.09	22.90	70.26	15.62	160.97	204.99	0.91	0.60

AI+B+PAM	1	Alternate	Yes	Yes	3.10	13.74	6.38	18.52	1.35	58.62	1.08	22.86	74.23	18.08	168.65	263.49	1.02	0.80
AI+B+PAM	2	Alternate	Yes	Yes	3.31	15.42	6.07	19.16	1.33	56.24	1.02	23.29	73.55	16.77	162.34	263.49	1.18	0.73
AI+B+PAM	3	Alternate	Yes	Yes	2.98	14.17	6.70	17.88	1.34	55.04	0.98	22.66	70.33	18.23	173.42	231.83	1.10	0.82
AI+B+PAM	4	Alternate	Yes	Yes	3.13	15.16	6.28	19.01	1.23	58.36	1.05	22.96	79.25	18.25	176.09	240.43	1.07	0.82

Table S2. Pot-level raw data for root traits, plant biomass, nutrient uptake, and plant nutrient concentration.

Treatment	Rep	Irrigation	Microbial inoculant	PAM	Phosphatase	Root_biomass	Root_length	Root_surface_area	Biomass	N_uptake	P_uptake	K_uptake	Plant_N	Plant_P	Plant_K
CI	1	Conventional	No	No	0.46	1.46	269.87	35.74	8.89	0.15	0.02	0.13	16.43	2.03	14.97
CI	2	Conventional	No	No	0.47	1.49	294.12	36.65	8.17	0.15	0.02	0.15	17.87	2.33	18.36
CI	3	Conventional	No	No	0.52	1.41	278.99	35.87	8.52	0.16	0.02	0.15	18.89	2.11	17.83
CI	4	Conventional	No	No	0.48	1.41	279.96	38.20	7.97	0.16	0.02	0.14	20.21	2.26	17.07
AI	1	Alternate	No	No	0.55	1.66	309.96	41.42	9.34	0.18	0.02	0.17	19.27	2.46	17.98
AI	2	Alternate	No	No	0.56	1.59	307.08	41.45	9.39	0.20	0.03	0.19	21.08	2.77	20.12
AI	3	Alternate	No	No	0.53	1.62	345.57	42.41	10.16	0.19	0.02	0.18	18.21	2.26	17.71
AI	4	Alternate	No	No	0.51	1.61	310.40	42.47	10.57	0.20	0.03	0.17	18.64	2.46	15.61
AI+B	1	Alternate	Yes	No	0.86	1.92	381.24	49.79	11.41	0.26	0.03	0.23	22.34	2.80	20.24
AI+B	2	Alternate	Yes	No	0.79	2.04	403.54	49.25	11.35	0.26	0.03	0.22	23.25	2.99	19.47
AI+B	3	Alternate	Yes	No	0.87	2.04	403.80	51.24	11.03	0.23	0.03	0.23	20.86	2.99	20.86
AI+B	4	Alternate	Yes	No	0.76	1.93	416.28	47.81	10.32	0.25	0.03	0.24	24.04	3.01	23.26

AI+PAM	1	Alternate	No	Yes	0.60	1.87	352.30	49.27	9.98	0.21	0.03	0.20	20.55	2.71	20.25
AI+PAM	2	Alternate	No	Yes	0.49	1.92	348.07	44.49	10.90	0.21	0.03	0.20	19.27	2.66	18.45
AI+PAM	3	Alternate	No	Yes	0.59	1.80	349.56	49.11	10.83	0.20	0.03	0.20	18.00	2.59	18.10
AI+PAM	4	Alternate	No	Yes	0.60	1.96	372.48	44.62	9.43	0.19	0.03	0.21	19.84	2.76	22.28
AI+B+PAM	1	Alternate	Yes	Yes	0.80	2.33	424.65	54.76	12.95	0.29	0.04	0.29	22.01	2.93	22.08
AI+B+PAM	2	Alternate	Yes	Yes	0.73	2.18	423.47	59.04	12.62	0.28	0.04	0.28	22.03	2.93	22.27
AI+B+PAM	3	Alternate	Yes	Yes	0.82	2.08	455.52	53.88	12.78	0.28	0.04	0.29	22.07	2.90	22.61
AI+B+PAM	4	Alternate	Yes	Yes	0.82	2.20	409.36	60.68	12.48	0.28	0.04	0.28	22.04	2.88	22.12

Table S3. Statistical analysis results of soil and plant indicators as affected by alternating brackish–freshwater irrigation, microbial inoculant and PAM application.

variable	Treatment	n	mean	variance	sd	se	letter
EC	CI	4	4.03375	0.039544	0.198857	0.099429	b
EC	AI	4	3.4415	0.039067	0.197654	0.098827	a
EC	AI+B	4	3.365	0.0225	0.15	0.075	a
EC	AI+PAM	4	3.185	0.01802	0.134239	0.067119	a
EC	AI+B+PAM	4	3.1305	0.018737	0.136883	0.068442	a
Na	CI	4	18.327	0.68346	0.826716	0.413358	b
Na	AI	4	15.356	0.378374	0.615121	0.307561	a
Na	AI+B	4	15.329	0.235479	0.485261	0.242631	a
Na	AI+PAM	4	14.734	0.638656	0.79916	0.39958	a
Na	AI+B+PAM	4	14.6215	0.639497	0.799686	0.399843	a
SAR	CI	4	8.183	0.040823	0.202046	0.101023	c
SAR	AI	4	7.0145	0.073172	0.270503	0.135251	b
SAR	AI+B	4	6.782	0.022909	0.151358	0.075679	ab
SAR	AI+PAM	4	6.619	0.059673	0.244281	0.122141	ab
SAR	AI+B+PAM	4	6.358	0.068467	0.261661	0.130831	a
SWC	CI	4	14.614	0.963218	0.981437	0.490718	a
SWC	AI	4	16.35125	0.355002	0.595821	0.29791	b
SWC	AI+B	4	16.657	0.067799	0.260382	0.130191	b
SWC	AI+PAM	4	18.77775	0.099183	0.314933	0.157467	c
SWC	AI+B+PAM	4	18.6425	0.332905	0.576979	0.28849	c
BD	AI	4	1.4065	0.001264	0.035557	0.017779	b
BD	AI+B	4	1.41125	0.001626	0.040327	0.020163	b
BD	AI+PAM	4	1.311	0.001435	0.037886	0.018943	a
BD	AI+B+PAM	4	1.31	0.00299	0.054681	0.02734	a
WSA	AI	4	45.9475	4.483591	2.117449	1.058725	a
WSA	AI+B	4	46.298	1.692973	1.301143	0.650572	a
WSA	AI+PAM	4	56.3025	2.289042	1.512958	0.756479	b
WSA	AI+B+PAM	4	57.064	2.955582	1.719181	0.85959	b
MWD	AI	4	0.787	0.004041	0.063566	0.031783	a
MWD	AI+B	4	0.78525	0.00186	0.043131	0.021565	a
MWD	AI+PAM	4	1.02175	0.004721	0.068709	0.034354	b
MWD	AI+B+PAM	4	1.03025	0.001576	0.039702	0.019851	b
FC	AI	4	19.7775	0.41925	0.647495	0.323747	a
FC	AI+B	4	19.839	0.212988	0.461506	0.230753	a
FC	AI+PAM	4	22.818	0.085021	0.291584	0.145792	b
FC	AI+B+PAM	4	22.94225	0.069954	0.264487	0.132244	b
AN	AI	4	65.0045	1.106347	1.05183	0.525915	a
AN	AI+B	4	74.4215	15.51512	3.938923	1.969462	b
AN	AI+PAM	4	70.83075	2.828654	1.68186	0.84093	ab
AN	AI+B+PAM	4	74.33925	13.61931	3.690435	1.845217	b
AP	AI	4	14.61375	0.228462	0.477977	0.238988	a
AP	AI+B	4	18.7045	0.051054	0.225951	0.112975	b
AP	AI+PAM	4	14.12975	2.687676	1.639413	0.819707	a

AP	AI+B+PAM	4	17.8315	0.510558	0.714534	0.357267	b
AK	AI	4	144.8953	33.34363	5.774394	2.887197	a
AK	AI+B	4	174.836	25.49098	5.048859	2.524429	c
AK	AI+PAM	4	160.738	11.96808	3.459492	1.729746	b
AK	AI+B+PAM	4	170.1228	36.39852	6.033118	3.016559	bc
MBC	AI	4	193.4603	67.95991	8.24378	4.12189	a
MBC	AI+B	4	258.2238	155.1078	12.45423	6.227114	b
MBC	AI+PAM	4	213.0088	79.38664	8.909918	4.454959	a
MBC	AI+B+PAM	4	249.8068	261.8311	16.1812	8.090598	b
Urease	AI	4	0.86175	0.000732	0.027048	0.013524	a
Urease	AI+B	4	1.17675	0.002912	0.053965	0.026983	b
Urease	AI+PAM	4	0.9555	0.001748	0.041813	0.020907	a
Urease	AI+B+PAM	4	1.092	0.004828	0.069484	0.034742	b
Phosphatase	AI	4	0.54025	0.000485	0.022021	0.01101	a
Phosphatase	AI+B	4	0.819	0.00285	0.053385	0.026693	b
Phosphatase	AI+PAM	4	0.57025	0.002973	0.054524	0.027262	a
Phosphatase	AI+B+PAM	4	0.79075	0.00186	0.043131	0.021565	b
Root_biomass	AI	4	1.62	0.000818	0.028601	0.0143	a
Root_biomass	AI+B	4	1.98325	0.004091	0.06396	0.03198	b
Root_biomass	AI+PAM	4	1.88625	0.004245	0.065153	0.032577	b
Root_biomass	AI+B+PAM	4	2.1995	0.010609	0.103	0.0515	c
Root_length	AI	4	318.254	333.7924	18.26999	9.134993	a
Root_length	AI+B	4	401.2138	212.6247	14.58166	7.290828	c
Root_length	AI+PAM	4	355.6008	129.64	11.38596	5.692979	b
Root_length	AI+B+PAM	4	428.2515	378.7265	19.4609	9.730449	c
Root_surface_area	AI	4	41.93875	0.335742	0.579433	0.289716	a
Root_surface_area	AI+B	4	49.5235	2.003884	1.415586	0.707793	b
Root_surface_area	AI+PAM	4	46.87275	7.164082	2.67658	1.33829	ab
Root_surface_area	AI+B+PAM	4	57.09	10.80787	3.287532	1.643766	c
Biomass	AI	4	9.867	0.35965	0.599708	0.299854	a
Biomass	AI+B	4	11.02725	0.253856	0.503841	0.25192	a
Biomass	AI+PAM	4	10.28175	0.502365	0.708777	0.354389	a
Biomass	AI+B+PAM	4	12.7075	0.041158	0.202875	0.101438	b
N_uptake	AI	4	0.19	7.93E-05	0.008907	0.004453	a
N_uptake	AI+B	4	0.24925	0.000208	0.014408	0.007204	b
N_uptake	AI+PAM	4	0.19925	0.000106	0.010275	0.005138	a
N_uptake	AI+B+PAM	4	0.28	1.93E-05	0.004397	0.002198	c
P_uptake	AI	4	0.0245	3.00E-06	0.001732	0.000866	a
P_uptake	AI+B	4	0.0325	1.67E-06	0.001291	0.000645	c
P_uptake	AI+PAM	4	0.0275	1.67E-06	0.001291	0.000645	b
P_uptake	AI+B+PAM	4	0.037	6.67E-07	0.000816	0.000408	d
K_uptake	AI	4	0.1755	0.000123	0.011091	0.005545	a
K_uptake	AI+B	4	0.2305	6.03E-05	0.007767	0.003884	c
K_uptake	AI+PAM	4	0.20225	3.36E-05	0.005795	0.002898	b
K_uptake	AI+B+PAM	4	0.283	3.27E-05	0.005715	0.002858	d
Plant_N	AI	4	19.29689	1.597836	1.264055	0.632028	a
Plant_N	AI+B	4	22.62372	1.865044	1.365666	0.682833	b
Plant_N	AI+PAM	4	19.41731	1.161359	1.077663	0.538832	a

Plant_N	AI+B+PAM	4	22.03431	0.000576	0.024002	0.012001	b
Plant_P	AI	4	2.488202	0.043369	0.208251	0.104126	a
Plant_P	AI+B	4	2.949086	0.009439	0.097154	0.048577	c
Plant_P	AI+PAM	4	2.678022	0.005405	0.07352	0.03676	ab
Plant_P	AI+B+PAM	4	2.911495	0.000642	0.025345	0.012673	bc
Plant_K	AI	4	17.8564	3.400462	1.844034	0.922017	a
Plant_K	AI+B	4	20.95729	2.691495	1.640578	0.820289	ab
Plant_K	AI+PAM	4	19.76878	3.696171	1.922543	0.961271	ab
Plant_K	AI+B+PAM	4	22.27001	0.05871	0.242302	0.121151	b

Table S4. Two-way ANOVA showing the significance of microbial inoculant, PAM and their interactive effects on measured soil and plant variables.

variable	factor	Df	Sum_Sq	Mean_Sq	F_value	P_value	significance
EC	Microbial inoculant	1	0.017161	0.017161	0.698141	0.419735	ns
EC	PAM	1	0.241081	0.241081	9.807616	0.008664	**
EC	Microbial inoculant:PAM	1	0.000484	0.000484	0.01969	0.890734	ns
EC	Residuals	12	0.294972	0.024581	NA	NA	ns
Na	Microbial inoculant	1	0.01946	0.01946	0.041142	0.842662	ns
Na	PAM	1	1.76757	1.76757	3.736924	0.077163	ns
Na	Microbial inoculant:PAM	1	0.00731	0.00731	0.015455	0.903121	ns
Na	Residuals	12	5.676017	0.473001	NA	NA	ns
SAR	Microbial inoculant	1	0.243542	0.243542	4.344682	0.059161	ns
SAR	PAM	1	0.67158	0.67158	11.98068	0.004706	**
SAR	Microbial inoculant:PAM	1	0.000812	0.000812	0.01449	0.906178	ns
SAR	Residuals	12	0.672663	0.056055	NA	NA	ns
SWC	Microbial inoculant	1	0.02907	0.02907	0.136019	0.718696	ns
SWC	PAM	1	19.46574	19.46574	91.07965	5.92E-07	***
SWC	Microbial inoculant:PAM	1	0.194481	0.194481	0.909971	0.358944	ns
SWC	Residuals	12	2.564667	0.213722	NA	NA	ns
BD	Microbial inoculant	1	1.41E-05	1.41E-05	0.007689	0.931573	ns
BD	PAM	1	0.038711	0.038711	21.16512	0.00061	***
BD	Microbial inoculant:PAM	1	3.31E-05	3.31E-05	0.018077	0.895275	ns
BD	Residuals	12	0.021948	0.001829	NA	NA	ns
WSA	Microbial inoculant	1	1.236544	1.236544	0.43307	0.522911	ns
WSA	PAM	1	446.0966	446.0966	156.2348	3.07E-08	***
WSA	Microbial inoculant:PAM	1	0.168921	0.168921	0.059161	0.811936	ns
WSA	Residuals	12	34.26356	2.855297	NA	NA	ns
MWD	Microbial inoculant	1	4.56E-05	4.56E-05	0.014941	0.904738	ns
MWD	PAM	1	0.23016	0.23016	75.47417	1.60E-06	***
MWD	Microbial inoculant:PAM	1	0.000105	0.000105	0.034452	0.855849	ns
MWD	Residuals	12	0.036594	0.00305	NA	NA	ns
FC	Microbial inoculant	1	0.034503	0.034503	0.175318	0.682825	ns
FC	PAM	1	37.74566	37.74566	191.794	9.65E-09	***
FC	Microbial inoculant:PAM	1	0.003938	0.003938	0.020008	0.889863	ns
FC	Residuals	12	2.361638	0.196803	NA	NA	ns

AN	Microbial inoculant	1	167.0686	167.0686	20.20822	0.000733	***
AN	PAM	1	32.99354	32.99354	3.99082	0.068932	ns
AN	Microbial inoculant:PAM	1	34.91037	34.91037	4.222676	0.062328	ns
AN	Residuals	12	99.20828	8.267357	NA	NA	ns
AP	Microbial inoculant	1	60.72306	60.72306	69.84179	2.40E-06	***
AP	PAM	1	1.841449	1.841449	2.117978	0.171236	ns
AP	Microbial inoculant:PAM	1	0.151321	0.151321	0.174045	0.683908	ns
AP	Residuals	12	10.43325	0.869437	NA	NA	ns
AK	Microbial inoculant	1	1546.495	1546.495	57.70439	6.36E-06	***
AK	PAM	1	123.8658	123.8658	4.621805	0.052658	ns
AK	Microbial inoculant:PAM	1	422.5491	422.5491	15.76658	0.001857	**
AK	Residuals	12	321.6036	26.8003	NA	NA	ns
MBC	Microbial inoculant	1	10314.74	10314.74	73.11716	1.89E-06	***
MBC	PAM	1	123.9103	123.9103	0.878352	0.367133	ns
MBC	Microbial inoculant:PAM	1	782.0692	782.0692	5.543784	0.036412	*
MBC	Residuals	12	1692.856	141.0714	NA	NA	ns
Urease	Microbial inoculant	1	0.203852	0.203852	79.78432	1.20E-06	***
Urease	PAM	1	8.10E-05	8.10E-05	0.031702	0.861654	ns
Urease	Microbial inoculant:PAM	1	0.031862	0.031862	12.47034	0.004136	**
Urease	Residuals	12	0.030661	0.002555	NA	NA	ns
Phosphatase	Microbial inoculant	1	0.249251	0.249251	122.0607	1.21E-07	***
Phosphatase	PAM	1	3.06E-06	3.06E-06	0.0015	0.969745	ns
Phosphatase	Microbial inoculant:PAM	1	0.003393	0.003393	1.66162	0.22168	ns
Phosphatase	Residuals	12	0.024504	0.002042	NA	NA	ns
Root_biomass	Microbial inoculant	1	0.457652	0.457652	92.62887	5.41E-07	***
Root_biomass	PAM	1	0.232806	0.232806	47.12001	1.74E-05	***
Root_biomass	Microbial inoculant:PAM	1	0.0025	0.0025	0.506	0.490472	ns
Root_biomass	Residuals	12	0.059289	0.004941	NA	NA	ns
Root_length	Microbial inoculant	1	24214.63	24214.63	91.82785	5.67E-07	***
Root_length	PAM	1	4145.364	4145.364	15.72024	0.001877	**
Root_length	Microbial inoculant:PAM	1	106.2755	106.2755	0.403023	0.537449	ns
Root_length	Residuals	12	3164.351	263.6959	NA	NA	ns
Root_surface_area	Microbial inoculant	1	316.9112	316.9112	62.40997	4.28E-06	***

Root_surface_area	PAM	1	156.2625	156.2625	30.77309	0.000126	***
Root_surface_area	Microbial inoculant:PAM	1	6.930056	6.930056	1.36475	0.265402	ns
Root_surface_area	Residuals	12	60.93472	5.077894	NA	NA	ns
Biomass	Microbial inoculant	1	12.8594	12.8594	44.45661	2.30E-05	***
Biomass	PAM	1	4.389025	4.389025	15.17343	0.002128	**
Biomass	Microbial inoculant:PAM	1	1.60149	1.60149	5.536561	0.036514	*
Biomass	Residuals	12	3.471087	0.289257	NA	NA	ns
N_uptake	Microbial inoculant	1	0.0196	0.0196	190.3683	1.01E-08	***
N_uptake	PAM	1	0.0016	0.0016	15.54027	0.001955	**
N_uptake	Microbial inoculant:PAM	1	0.000462	0.000462	4.48968	0.055645	ns
N_uptake	Residuals	12	0.001236	0.000103	NA	NA	ns
P_uptake	Microbial inoculant	1	0.000306	0.000306	175	1.62E-08	***
P_uptake	PAM	1	5.62E-05	5.62E-05	32.14286	0.000104	***
P_uptake	Microbial inoculant:PAM	1	2.25E-06	2.25E-06	1.285714	0.278984	ns
P_uptake	Residuals	12	2.10E-05	1.75E-06	NA	NA	ns
K_uptake	Microbial inoculant	1	0.018428	0.018428	295.3412	8.13E-10	***
K_uptake	PAM	1	0.006281	0.006281	100.6568	3.46E-07	***
K_uptake	Microbial inoculant:PAM	1	0.000663	0.000663	10.62671	0.00683	**
K_uptake	Residuals	12	0.000749	6.24E-05	NA	NA	ns
Plant_N	Microbial inoculant	1	35.32904	35.32904	30.55607	0.00013	***
Plant_N	PAM	1	0.219953	0.219953	0.190237	0.670463	ns
Plant_N	Microbial inoculant:PAM	1	0.50385	0.50385	0.43578	0.521636	ns
Plant_N	Residuals	12	13.87444	1.156204	NA	NA	ns
Plant_P	Microbial inoculant	1	0.482132	0.482132	32.76748	9.54E-05	***
Plant_P	PAM	1	0.023174	0.023174	1.574964	0.233378	ns
Plant_P	Microbial inoculant:PAM	1	0.051715	0.051715	3.514772	0.085364	ns
Plant_P	Residuals	12	0.176565	0.014714	NA	NA	ns
Plant_K	Microbial inoculant	1	31.38359	31.38359	12.7487	0.003849	**
Plant_K	PAM	1	10.40129	10.40129	4.225231	0.06226	ns
Plant_K	Microbial inoculant:PAM	1	0.359591	0.359591	0.146074	0.708999	ns
Plant_K	Residuals	12	29.54051	2.461709	NA	NA	ns

Table S5. Pot-level input matrix used for the exploratory PLS-PM analysis.

PAM_app	Bio_app	SWC	WSA	AK	MBC	Root_biomass	Root_surface_area	Biomass	K_uptake
0	0	16.71	48.727	142.068	194.35	1.66	41.423	9.343	0.168
0	0	16.71	44.313	138.718	201.073	1.593	41.452	9.394	0.189
0	0	15.468	46.47	151.98	181.827	1.618	42.41	10.161	0.18
0	0	16.517	44.28	146.815	196.591	1.609	42.47	10.57	0.165
0	1	16.948	47.812	176.936	247.26	1.923	49.794	11.414	0.231
0	1	16.338	45.126	167.528	268.919	2.036	49.253	11.353	0.221
0	1	16.762	46.95	179.036	247.618	2.041	51.236	11.026	0.23
0	1	16.58	45.304	175.844	269.098	1.933	47.811	10.316	0.24
1	0	18.735	58.095	165.53	220.722	1.869	49.273	9.975	0.202
1	0	18.366	55.91	158.608	220.722	1.917	44.489	10.896	0.201
1	0	19.112	54.48	157.848	205.598	1.804	49.106	10.831	0.196
1	0	18.898	56.725	160.966	204.993	1.955	44.623	9.425	0.21
1	1	18.524	58.618	168.647	263.486	2.333	54.76	12.95	0.286
1	1	19.156	56.238	162.339	263.486	2.179	59.04	12.62	0.281
1	1	17.878	55.041	173.418	231.826	2.083	53.88	12.78	0.289
1	1	19.012	58.359	176.087	240.429	2.203	60.68	12.48	0.276

Table S6. Standardized path coefficients and bootstrap confidence intervals of the exploratory PLS-PM model.

Path	Beta	Boot_mean	Boot_SE	CI_2.5	CI_97.5	Significance
PAM → Physical conditioning	0.969759	0.971102	0.011734	0.945674	0.990461	***
Microbial inoculant → Biological stimulation	0.905169	0.909201	0.035436	0.82629	0.971045	***
Physical conditioning → Root development	0.452422	0.453001	0.141557	0.230381	0.776597	**
Biological stimulation → Root development	0.718287	0.74979	0.1137	0.554422	0.964748	*
Root development → Plant performance	0.933064	0.933586	0.028466	0.859729	0.976136	**

Code S1. R scripts for statistical analysis, figure generation, and exploratory PLS-PM analysis.

```
#### Clear workspace ####
rm(list = ls())

#### Load required packages ####
packages <- c(
  "tidyverse",
  "ggplot2",
  "patchwork",
  "emmeans",
  "multcomp",
  "multcompView"
)

new_packages <- packages[!(packages %in% installed.packages()[, "Package"])]
if (length(new_packages) > 0) install.packages(new_packages)

lapply(packages, library, character.only = TRUE)

#### Global graphical parameters ####

font_family <- "Arial"

axis_line_width <- 0.8
errorbar_width  <- 0.8
bar_border_width <- 0.5

axis_title_size <- 9
axis_text_size  <- 8
legend_text_size <- 8
legend_title_size <- 8
strip_text_size <- 9
tag_size <- 11
sig_text_size <- 3
anova_text_size <- 2.0

dpi_sci <- 600

theme_sci <- function(base_size = 8, base_family = font_family) {
  theme_classic(base_size = base_size, base_family = base_family) +
    theme(
```

```
    text = element_text(family = base_family, color = "black"),

    axis.title = element_text(size = axis_title_size, color = "black"),
    axis.text = element_text(size = axis_text_size, color = "black"),
    axis.line = element_line(linewidth = axis_line_width, color = "black"),
    axis.ticks = element_line(linewidth = axis_line_width, color = "black"),
    axis.ticks.length = unit(2, "mm"),

    legend.title = element_text(size = legend_title_size),
    legend.text = element_text(size = legend_text_size),
    legend.key.size = unit(4, "mm"),
    legend.position = "top",

    strip.background = element_blank(),
    strip.text = element_text(size = strip_text_size, face = "bold"),

    plot.title = element_text(size = 10, face = "bold", hjust = 0),
    plot.tag = element_text(size = tag_size, face = "bold"),

    plot.margin = margin(5, 5, 5, 5)
  )
}
pal_5 <- c(
  "CI" = "#BDBDBD",
  "AI" = "#4E79A7",
  "AI+B" = "#59A14F",
  "AI+PAM" = "#F28E2B",
  "AI+B+PAM" = "#E15759"
)

pal_factorial <- c(
  "CK" = "#4E79A7",
  "B" = "#59A14F",
  "PAM" = "#F28E2B",
  "B+PAM" = "#E15759"
)


#### TIFF  ####

save_sci <- function(plot, filename, width_mm = 180, height_mm = 120, dpi = 600) {
  ggsave(
```

```
    filename = filename,
    plot = plot,
    width = width_mm / 25.4,
    height = height_mm / 25.4,
    dpi = dpi,
    compression = "lzw"
  )
}
p_star <- function(p) {
  dplyr::case_when(
    is.na(p) ~ "ns",
    p < 0.001 ~ "***",
    p < 0.01  ~ "**",
    p < 0.05  ~ "*",
    TRUE ~ "ns"
  )
}

setwd("E:/BaiduSyncdisk")

main_df <- read.csv(
  "cotton_pot_main_data.csv",
  header = TRUE,
  stringsAsFactors = FALSE,
  check.names = FALSE
)

names(main_df) <- trimws(names(main_df))
names(main_df) <- gsub("\ufeff", "", names(main_df), fixed = TRUE)

main_df <- main_df[, names(main_df) != "", drop = FALSE]

if (any(duplicated(names(main_df)))) {
  print("发现重复列名：")
  print(names(main_df)[duplicated(names(main_df)) | duplicated(names(main_df), fromLast = TRUE)])
  main_df <- main_df[, !duplicated(names(main_df)), drop = FALSE]
}

if ("Biofertillizer" %in% names(main_df) & !("Microbial inoculant" %in% names(main_df))) {
  main_df <- main_df %>%
    dplyr::rename(Microbial inoculant = Biofertillizer)
```

```
}

main_df <- main_df %>%
  dplyr::mutate(
    Treatment = trimws(as.character(Treatment)),
    Irrigation = trimws(as.character(Irrigation)),
    Microbial inoculant = trimws(as.character(Microbial inoculant)),
    PAM = trimws(as.character(PAM)),

    Microbial inoculant = dplyr::case_when(
      Microbial inoculant %in% c("Yes", "YES", "yes", "Y", "1", "+", "有") ~ "Yes",
      Microbial inoculant %in% c("No", "NO", "no", "N", "0", "-", "无") ~ "No",
      Microbial inoculant %in% c("NA", "Na", "na", "", "/", "—") ~ NA_character_,
      TRUE ~ Microbial inoculant
    ),

    PAM = dplyr::case_when(
      PAM %in% c("Yes", "YES", "yes", "Y", "1", "+", "有") ~ "Yes",
      PAM %in% c("No", "NO", "no", "N", "0", "-", "无") ~ "No",
      PAM %in% c("NA", "Na", "na", "", "/", "—") ~ NA_character_,
      TRUE ~ PAM
    ),

    Treatment = factor(Treatment, levels = c("P1", "P2", "P3", "P4", "P5")),
    Irrigation = factor(Irrigation, levels = c("Conventional", "Alternate")),
    Microbial inoculant = factor(Microbial inoculant, levels = c("No", "Yes")),
    PAM = factor(PAM, levels = c("No", "Yes")),

    # Fig.2
    Treatment_label_full = dplyr::case_when(
      Treatment == "P1" ~ "CI",
      Treatment == "P2" ~ "AI",
      Treatment == "P3" ~ "AI+B",
      Treatment == "P4" ~ "AI+PAM",
      Treatment == "P5" ~ "AI+B+PAM"
    ),
    Treatment_label_full = factor(
      Treatment_label_full,
      levels = c("CI", "AI", "AI+B", "AI+PAM", "AI+B+PAM")
    ),

    # Fig.3-Fig.7
```

```
    Treatment_label_factorial = dplyr::case_when(
      Treatment == "P2" ~ "CK",
      Treatment == "P3" ~ "B",
      Treatment == "P4" ~ "PAM",
      Treatment == "P5" ~ "B+PAM",
      TRUE ~ NA_character_
    ),
    Treatment_label_factorial = factor(
      Treatment_label_factorial,
      levels = c("CK", "B", "PAM", "B+PAM")
    )
  )

print(names(main_df))
print(table(main_df$Treatment, useNA = "ifany"))
print(table(main_df$Treatment_label_full, useNA = "ifany"))
print(table(main_df$Treatment_label_factorial, useNA = "ifany"))
print(table(main_df$Treatment, main_df$Microbial inoculant, useNA = "ifany"))
print(table(main_df$Treatment, main_df$PAM, useNA = "ifany"))

check_df <- main_df %>%
  dplyr::filter(Treatment %in% c("P2", "P3", "P4", "P5"))

print(with(check_df, table(Microbial inoculant, PAM, useNA = "ifany")))

plot_all_rep_bar_with_factorial <- function(data, y, ylab) {

  y_var <- rlang::sym(y)

  data_all <- data %>%
    dplyr::mutate(
      Treatment = factor(Treatment, levels = c("P1", "P2", "P3", "P4", "P5")),
      Treatment_label_full = factor(
        Treatment_label_full,
        levels = c("CI", "AI", "AI+B", "AI+PAM", "AI+B+PAM")
      ),
      value = as.numeric(!!y_var)
    ) %>%
    dplyr::filter(!is.na(value))

  # ANOVA
  model_oneway <- stats::aov(value ~ Treatment_label_full, data = data_all)
```

```
em_oneway <- emmeans::emmeans(model_oneway, ~ Treatment_label_full)

letters_df <- multcomp::cld(
  em_oneway,
  Letters = letters,
  adjust = "Tukey"
) %>%
  as.data.frame() %>%
  dplyr::mutate(
    Treatment_label_full = factor(
      Treatment_label_full,
      levels = c("CI", "AI", "AI+B", "AI+PAM", "AI+B+PAM")
    ),
    letter = stringr::str_trim(.group)
  ) %>%
  dplyr::select(Treatment_label_full, letter)

summary_df <- data_all %>%
  dplyr::group_by(Treatment_label_full) %>%
  dplyr::summarise(
    mean = mean(value, na.rm = TRUE),
    se = sd(value, na.rm = TRUE) / sqrt(dplyr::n()),
    .groups = "drop"
  ) %>%
  dplyr::left_join(letters_df, by = "Treatment_label_full")

# ANOVA
data_factorial <- data_all %>%
  dplyr::filter(Treatment %in% c("P2", "P3", "P4", "P5")) %>%
  dplyr::mutate(
    Microbial inoculant = factor(Microbial inoculant, levels = c("No", "Yes")),
    PAM = factor(PAM, levels = c("No", "Yes"))
  ) %>%
  dplyr::filter(!is.na(Microbial inoculant), !is.na(PAM))

model_factorial <- stats::aov(value ~ Microbial inoculant * PAM, data = data_factorial)
anova_tab <- stats::anova(model_factorial)

p_bio <- anova_tab$`Pr(>F)`[1]
p_pam <- anova_tab$`Pr(>F)`[2]
p_int <- anova_tab$`Pr(>F)`[3]
```

```
p_text <- paste0(
  "Bio: ", p_star(p_bio), "; PAM: ", p_star(p_pam), "\n",
  "Bio×PAM: ", p_star(p_int)
)

ymax <- max(summary_df$mean + summary_df$se, na.rm = TRUE)
letter_top <- max(summary_df$mean + summary_df$se + ymax * 0.05, na.rm = TRUE)
y_lim <- max(ymax * 1.25, letter_top * 1.12)

label_x <- 5.55
label_y <- y_lim * 0.96

ggplot(summary_df, aes(x = Treatment_label_full, y = mean, fill = Treatment_label_full)) +
  geom_col(
    width = 0.62,
    color = "black",
    linewidth = bar_border_width
  ) +
  geom_errorbar(
    aes(ymin = mean - se, ymax = mean + se),
    width = 0.18,
    linewidth = errorbar_width
  ) +
  geom_text(
    aes(y = mean + se + ymax * 0.045, label = letter),
    size = sig_text_size,
    family = font_family
  ) +
  annotate(
    "text",
    x = label_x,
    y = label_y,
    label = p_text,
    hjust = 1,
    vjust = 1,
    size = anova_text_size,
    family = font_family
  ) +
  scale_fill_manual(values = pal_5) +
  scale_x_discrete(expand = expansion(add = c(0.5, 1.05))) +
  labs(x = NULL, y = ylab) +
  coord_cartesian(ylim = c(0, y_lim), clip = "off") +
```

```
  theme_sci() +
  theme(
    legend.position = "none",
    axis.text.x = element_text(size = 7, angle = 25, hjust = 1),
    plot.margin = margin(5, 18, 5, 5)
  )
}

plot_factorial_rep_bar <- function(data, y, ylab) {

 y_var <- rlang::sym(y)

 data_sub <- data %>%
  dplyr::filter(Treatment %in% c("P2", "P3", "P4", "P5")) %>%
  dplyr::mutate(
    Treatment_label_factorial = factor(
     Treatment_label_factorial,
     levels = c("CK", "B", "PAM", "B+PAM")
    ),
    Microbial inoculant = factor(Microbial inoculant, levels = c("No", "Yes")),
    PAM = factor(PAM, levels = c("No", "Yes")),
    value = as.numeric(!!y_var)
  ) %>%
  dplyr::filter(!is.na(value), !is.na(Microbial inoculant), !is.na(PAM))

 summary_df <- data_sub %>%
  dplyr::group_by(Treatment_label_factorial) %>%
  dplyr::summarise(
    mean = mean(value, na.rm = TRUE),
    se = sd(value, na.rm = TRUE) / sqrt(dplyr::n()),
    .groups = "drop"
  )

 model <- stats::aov(value ~ Microbial inoculant * PAM, data = data_sub)
 anova_tab <- stats::anova(model)

 p_bio <- anova_tab$`Pr(>F)`[1]
 p_pam <- anova_tab$`Pr(>F)`[2]
 p_int <- anova_tab$`Pr(>F)`[3]

 p_text <- paste0(
  "Bio: ", p_star(p_bio), "; PAM: ", p_star(p_pam), "\n",
```

```
  "Bio×PAM: ", p_star(p_int)
)

em <- emmeans::emmeans(model, ~ Microbial inoculant * PAM)

cld_df <- multcomp::cld(
  em,
  Letters = letters,
  adjust = "sidak"
) %>%
  as.data.frame() %>%
  dplyr::mutate(
    Treatment_label_factorial = dplyr::case_when(
      Microbial inoculant == "No"  & PAM == "No"  ~ "CK",
      Microbial inoculant == "Yes" & PAM == "No"  ~ "B",
      Microbial inoculant == "No"  & PAM == "Yes" ~ "PAM",
      Microbial inoculant == "Yes" & PAM == "Yes" ~ "B+PAM"
    ),
    Treatment_label_factorial = factor(
      Treatment_label_factorial,
      levels = c("CK", "B", "PAM", "B+PAM")
    ),
    letter = stringr::str_trim(.group)
  ) %>%
  dplyr::select(Treatment_label_factorial, letter)

summary_df <- summary_df %>%
  dplyr::left_join(cld_df, by = "Treatment_label_factorial")

ymax <- max(summary_df$mean + summary_df$se, na.rm = TRUE)
letter_top <- max(summary_df$mean + summary_df$se + ymax * 0.05, na.rm = TRUE)
y_lim <- max(ymax * 1.32, letter_top * 1.18)

label_x <- 4.55
label_y <- y_lim * 0.97

ggplot(summary_df, aes(x = Treatment_label_factorial, y = mean, fill = Treatment_label_factorial))
+
  geom_col(
    width = 0.62,
    color = "black",
    linewidth = bar_border_width
```

```
    ) +
    geom_errorbar(
      aes(ymin = mean - se, ymax = mean + se),
      width = 0.18,
      linewidth = errorbar_width
    ) +
    geom_text(
      aes(y = mean + se + ymax * 0.045, label = letter),
      size = sig_text_size,
      family = font_family
    ) +
    annotate(
      "text",
      x = label_x,
      y = label_y,
      label = p_text,
      hjust = 1,
      vjust = 1,
      size = anova_text_size,
      family = font_family
    ) +
    scale_fill_manual(values = pal_factorial) +
    scale_x_discrete(expand = expansion(add = c(0.5, 1.05))) +
    labs(x = NULL, y = ylab) +
    coord_cartesian(ylim = c(0, y_lim), clip = "off") +
    theme_sci() +
    theme(
      legend.position = "none",
      axis.text.x = element_text(size = 8, angle = 0, hjust = 0.5),
      plot.margin = margin(5, 18, 5, 5)
    )
}


if (!dir.exists("result_tables")) {
  dir.create("result_tables")
}

export_fig2_stats <- function(data, vars, fig_name = "Fig2") {

  all_summary_list <- list()
  factorial_list <- list()
```

```
for (v in vars) {

  data_all <- data %>%
    dplyr::mutate(
      Treatment_label_full = factor(
        Treatment_label_full,
        levels = c("CI", "AI", "AI+B", "AI+PAM", "AI+B+PAM")
      ),
      value = as.numeric(.data[[v]])
    ) %>%
    dplyr::filter(!is.na(value))

  model_oneway <- stats::aov(value ~ Treatment_label_full, data = data_all)
  em_oneway <- emmeans::emmeans(model_oneway, ~ Treatment_label_full)

  letters_df <- multcomp::cld(
    em_oneway,
    Letters = letters,
    adjust = "sidak"
  ) %>%
    as.data.frame() %>%
    dplyr::mutate(
      Treatment_label_full = factor(
        Treatment_label_full,
        levels = c("CI", "AI", "AI+B", "AI+PAM", "AI+B+PAM")
      ),
      letter = stringr::str_trim(.group)
    ) %>%
    dplyr::select(Treatment_label_full, letter)

  summary_df <- data_all %>%
    dplyr::group_by(Treatment_label_full) %>%
    dplyr::summarise(
      variable = v,
      n = dplyr::n(),
      mean = mean(value, na.rm = TRUE),
      variance = var(value, na.rm = TRUE),
      sd = sd(value, na.rm = TRUE),
      se = sd(value, na.rm = TRUE) / sqrt(dplyr::n()),
      .groups = "drop"
    ) %>%
```

```
    dplyr::left_join(letters_df, by = "Treatment_label_full") %>%
    dplyr::rename(Treatment = Treatment_label_full) %>%
    dplyr::select(variable, Treatment, n, mean, variance, sd, se, letter)

  all_summary_list[[v]] <- summary_df

  data_factorial <- data_all %>%
    dplyr::filter(Treatment %in% c("P2", "P3", "P4", "P5")) %>%
    dplyr::mutate(
      Microbial inoculant = factor(Microbial inoculant, levels = c("No", "Yes")),
      PAM = factor(PAM, levels = c("No", "Yes"))
    ) %>%
    dplyr::filter(!is.na(Microbial inoculant), !is.na(PAM))

  model_factorial <- stats::aov(value ~ Microbial inoculant * PAM, data = data_factorial)
  anova_tab <- stats::anova(model_factorial)

  factorial_df <- data.frame(
    variable = v,
    factor = rownames(anova_tab),
    Df = anova_tab$Df,
    Sum_Sq = anova_tab$`Sum Sq`,
    Mean_Sq = anova_tab$`Mean Sq`,
    F_value = anova_tab$`F value`,
    P_value = anova_tab$`Pr(>F)`
  ) %>%
    dplyr::mutate(significance = p_star(P_value))

  factorial_list[[v]] <- factorial_df
}

all_summary <- dplyr::bind_rows(all_summary_list)
factorial_summary <- dplyr::bind_rows(factorial_list)

write.csv(
  all_summary,
  paste0("result_tables/", fig_name, "_treatment_mean_variance_letters.csv"),
  row.names = FALSE
)

write.csv(
  factorial_summary,
```

```
    paste0("result_tables/", fig_name, "_P2_P5_two_way_ANOVA.csv"),
    row.names = FALSE
  )

  return(list(treatment_summary = all_summary, factorial_anova = factorial_summary))
}


export_factorial_stats <- function(data, vars, fig_name = "Fig") {

  treatment_summary_list <- list()
  factorial_list <- list()

  for (v in vars) {
    data_sub <- data %>%
      dplyr::filter(Treatment %in% c("P2", "P3", "P4", "P5")) %>%
      dplyr::mutate(
        Treatment_label_factorial = factor(
          Treatment_label_factorial,
          levels = c("CK", "B", "PAM", "B+PAM")
        ),
        Microbial inoculant = factor(Microbial inoculant, levels = c("No", "Yes")),
        PAM = factor(PAM, levels = c("No", "Yes")),
        value = as.numeric(.data[[v]])
      ) %>%
      dplyr::filter(!is.na(value), !is.na(Microbial inoculant), !is.na(PAM))

    model <- stats::aov(value ~ Microbial inoculant * PAM, data = data_sub)
    anova_tab <- stats::anova(model)

    em <- emmeans::emmeans(model, ~ Microbial inoculant * PAM)

    cld_df <- multcomp::cld(
      em,
      Letters = letters,
      adjust = "sidak"
    ) %>%
      as.data.frame() %>%
      dplyr::mutate(
        Treatment_label_factorial = dplyr::case_when(
          Microbial inoculant == "No"  & PAM == "No"  ~ "CK",
          Microbial inoculant == "Yes" & PAM == "No"  ~ "B",
```

```
      Microbial inoculant == "No"  & PAM == "Yes" ~ "PAM",
      Microbial inoculant == "Yes" & PAM == "Yes" ~ "B+PAM"
    ),
    Treatment_label_factorial = factor(
      Treatment_label_factorial,
      levels = c("CK", "B", "PAM", "B+PAM")
    ),
    letter = stringr::str_trim(.group)
  ) %>%
  dplyr::select(Treatment_label_factorial, letter)

 treatment_summary <- data_sub %>%
  dplyr::group_by(Treatment_label_factorial) %>%
  dplyr::summarise(
    variable = v,
    n = dplyr::n(),
    mean = mean(value, na.rm = TRUE),
    variance = var(value, na.rm = TRUE),
    sd = sd(value, na.rm = TRUE),
    se = sd(value, na.rm = TRUE) / sqrt(dplyr::n()),
    .groups = "drop"
  ) %>%
  dplyr::left_join(cld_df, by = "Treatment_label_factorial") %>%
  dplyr::rename(Treatment = Treatment_label_factorial) %>%
  dplyr::select(variable, Treatment, n, mean, variance, sd, se, letter)

 factorial_df <- data.frame(
  variable = v,
  factor = rownames(anova_tab),
  Df = anova_tab$Df,
  Sum_Sq = anova_tab$`Sum Sq`,
  Mean_Sq = anova_tab$`Mean Sq`,
  F_value = anova_tab$`F value`,
  P_value = anova_tab$`Pr(>F)`
 ) %>%
  dplyr::mutate(significance = p_star(P_value))

 treatment_summary_list[[v]] <- treatment_summary
 factorial_list[[v]] <- factorial_df
}

treatment_summary_all <- dplyr::bind_rows(treatment_summary_list)
```

```
  factorial_summary_all <- dplyr::bind_rows(factorial_list)

  write.csv(
    treatment_summary_all,
    paste0("result_tables/", fig_name, "_treatment_mean_variance_letters.csv"),
    row.names = FALSE
  )

  write.csv(
    factorial_summary_all,
    paste0("result_tables/", fig_name, "_two_way_ANOVA.csv"),
    row.names = FALSE
  )

  return(list(treatment_summary = treatment_summary_all, factorial_anova = 
factorial_summary_all))
}

fig2_vars <- c("EC", "Na", "SAR", "SWC")
fig3_vars <- c("BD", "WSA", "MWD", "FC")
fig4_vars <- c("AN", "AP", "AK", "MBC", "Urease", "Phosphatase")
fig5_vars <- c("Root_biomass", "Root_length", "Root_surface_area")
fig6_vars <- c("Biomass", "N_uptake", "P_uptake", "K_uptake")
fig7_vars <- c("Plant_N", "Plant_P", "Plant_K")

fig2_stats <- export_fig2_stats(main_df, fig2_vars, "Fig2_root_zone_water_salt")
fig3_stats <- export_factorial_stats(main_df, fig3_vars, "Fig3_PAM_structure_water_retention")
fig4_stats <- export_factorial_stats(main_df, fig4_vars, "Fig4_microbial
inoculant_fertility_stabilization")
fig5_stats <- export_factorial_stats(main_df, fig5_vars, "Fig5_root_growth_response")
fig6_stats <- export_factorial_stats(main_df, fig6_vars, "Fig6_biomass_nutrient_uptake")
fig7_stats <- export_factorial_stats(main_df, fig7_vars, "Fig7_PLANT_NPK")

all_treatment_summary <- dplyr::bind_rows(
  fig2_stats$treatment_summary,
  fig3_stats$treatment_summary,
  fig4_stats$treatment_summary,
  fig5_stats$treatment_summary,
  fig6_stats$treatment_summary,
  fig7_stats$treatment_summary
)
```

```
all_factorial_anova <- dplyr::bind_rows(
  fig2_stats$factorial_anova,
  fig3_stats$factorial_anova,
  fig4_stats$factorial_anova,
  fig5_stats$factorial_anova,
  fig6_stats$factorial_anova,
  fig7_stats$factorial_anova
)

write.csv(
  all_treatment_summary,
  "result_tables/All_figures_treatment_mean_variance_letters.csv",
  row.names = FALSE
)

write.csv(
  all_factorial_anova,
  "result_tables/All_figures_two_way_ANOVA.csv",
  row.names = FALSE
)

p_ec  <- plot_all_rep_bar_with_factorial(main_df, "EC",  "EC (dS m\u207B\u00B9)")
p_na  <- plot_all_rep_bar_with_factorial(main_df, "Na",  "Na\u207A (mmol kg\u207B\u00B9)")
p_sar <- plot_all_rep_bar_with_factorial(main_df, "SAR", "SAR")
p_swc <- plot_all_rep_bar_with_factorial(main_df, "SWC", "Soil water content (%)")

fig2 <- (p_ec | p_na) / (p_sar | p_swc) +
  patchwork::plot_annotation(tag_levels = "a") &
  theme(plot.tag = element_text(size = tag_size, face = "bold", family = font_family))

fig2
save_sci(fig2, "Fig2_root_zone_water_salt.tiff", height_mm = 125)

p_bd  <- plot_factorial_rep_bar(main_df, "BD",  "Bulk density (g cm\u207B\u00B3)")
p_wsa <- plot_factorial_rep_bar(main_df, "WSA", "Water-stable aggregates (%)")
p_mwd <- plot_factorial_rep_bar(main_df, "MWD", "MWD (mm)")
p_fc  <- plot_factorial_rep_bar(main_df, "FC",  "Field capacity (%)")

fig3 <- (p_bd | p_wsa) / (p_mwd | p_fc) +
  patchwork::plot_annotation(tag_levels = "a") &
  theme(plot.tag = element_text(size = tag_size, face = "bold", family = font_family))
```

```
fig3
save_sci(fig3, "Fig3_PAM_structure_water_retention.tiff", height_mm = 125)

p_an  <- plot_factorial_rep_bar(main_df, "AN", "Available N (mg kg\u207B\u00B9)")
p_ap  <- plot_factorial_rep_bar(main_df, "AP", "Available P (mg kg\u207B\u00B9)")
p_ak  <- plot_factorial_rep_bar(main_df, "AK", "Available K (mg kg\u207B\u00B9)")
p_mbc <- plot_factorial_rep_bar(main_df, "MBC", "MBC (mg kg\u207B\u00B9)")
p_ur  <- plot_factorial_rep_bar(main_df, "Urease", "Urease activity")
p_ph  <- plot_factorial_rep_bar(main_df, "Phosphatase", "Phosphatase activity")

fig4 <- (p_an | p_ap | p_ak) / (p_mbc | p_ur | p_ph) +
  patchwork::plot_annotation(tag_levels = "a") &
  theme(plot.tag = element_text(size = tag_size, face = "bold", family = font_family))

fig4
save_sci(fig4, "Fig4_microbial inoculant_fertility_stabilization.tiff", height_mm = 125)

p_rb  <- plot_factorial_rep_bar(main_df, "Root_biomass", "Root biomass (g plant\u207B\u00B9)")
p_rl  <- plot_factorial_rep_bar(main_df, "Root_length", "Root length (cm plant\u207B\u00B9)")
p_rsa  <-  plot_factorial_rep_bar(main_df,  "Root_surface_area",  "Root  surface  area  (cm\u00B2
plant\u207B\u00B9)")

fig5 <- p_rb | p_rl | p_rsa +
  patchwork::plot_annotation(tag_levels = "a") &
  theme(plot.tag = element_text(size = tag_size, face = "bold", family = font_family))

fig5
save_sci(fig5, "Fig5_root_growth_response.tiff", height_mm = 65)

p_bio <- plot_factorial_rep_bar(main_df, "Biomass", "Biomass (g plant\u207B\u00B9)")
p_nu  <- plot_factorial_rep_bar(main_df, "N_uptake", "N uptake (g plant\u207B\u00B9)")
p_pu  <- plot_factorial_rep_bar(main_df, "P_uptake", "P uptake (g plant\u207B\u00B9)")
p_ku  <- plot_factorial_rep_bar(main_df, "K_uptake", "K uptake (g plant\u207B\u00B9)")

fig6 <- (p_bio | p_nu) / (p_pu | p_ku) +
  patchwork::plot_annotation(tag_levels = "a") &
  theme(plot.tag = element_text(size = tag_size, face = "bold", family = font_family))

fig6
save_sci(fig6, "Fig6_biomass_nutrient_uptake.tiff", height_mm = 125)

p_pN <- plot_factorial_rep_bar(main_df, "Plant_N", "Plant N (g kg\u207B\u00B9)")
```

```r
p_pP <- plot_factorial_rep_bar(main_df, "Plant_P", "Plant P (g kg\u207B\u00B9)")
p_pK <- plot_factorial_rep_bar(main_df, "Plant_K", "Plant K (g kg\u207B\u00B9)")

fig6_5 <- (p_pN | p_pP | p_pK) +
  patchwork::plot_annotation(tag_levels = "a") &
  theme(plot.tag = element_text(size = tag_size, face = "bold", family = font_family))

fig6_5
save_sci(fig6_5, "Fig6_5_plant_NPK.tiff", height_mm = 65)

heat_vars <- c(
  "EC", "SAR", "SWC",
  "BD", "WSA", "MWD", "FC",
  "AN", "AP", "AK", "MBC", "Urease", "Phosphatase",
  "Root_length", "Root_surface_area",
  "Biomass", "N_uptake", "P_uptake", "K_uptake"
)

indicator_order <- c(
  "EC", "SAR", "SWC",
  "BD", "WSA", "MWD", "FC",
  "AN", "AP", "AK", "MBC", "Urease", "Phosphatase",
  "Root_length", "Root_surface_area",
  "Biomass", "N_uptake", "P_uptake", "K_uptake"
)

heat_df <- main_df %>%
  dplyr::filter(Treatment %in% c("P2", "P3", "P4", "P5")) %>%
  dplyr::select(Treatment_label_factorial, dplyr::any_of(heat_vars)) %>%
  dplyr::mutate(
    Treatment_label_factorial = factor(
      Treatment_label_factorial,
      levels = c("CK", "B", "PAM", "B+PAM")
    ),
    dplyr::across(.cols = dplyr::any_of(heat_vars), .fns = as.numeric)
  ) %>%
  dplyr::group_by(Treatment_label_factorial) %>%
  dplyr::summarise(
    dplyr::across(.cols = dplyr::any_of(heat_vars), .fns = ~ mean(.x, na.rm = TRUE)),
    .groups = "drop"
  )
```

```r
negative_vars <- c("EC", "SAR", "BD")
heat_df_reverse <- heat_df

for (v in negative_vars) {
  if (v %in% names(heat_df_reverse)) {
    heat_df_reverse[[v]] <- -heat_df_reverse[[v]]
  }
}

heat_scaled <- heat_df_reverse %>%
  dplyr::mutate(
    dplyr::across(.cols = dplyr::any_of(heat_vars), .fns = ~ as.numeric(scale(.x)))
  )

heat_long <- heat_scaled %>%
  tidyr::pivot_longer(
    cols = dplyr::any_of(heat_vars),
    names_to = "Indicator",
    values_to = "Z"
  ) %>%
  dplyr::mutate(
    Treatment_label_factorial = factor(
      Treatment_label_factorial,
      levels = c("CK", "B", "PAM", "B+PAM")
    ),
    Indicator = factor(Indicator, levels = indicator_order)
  )

fig7 <- ggplot(heat_long, aes(x = Indicator, y = Treatment_label_factorial, fill = Z)) +
  geom_tile(color = "white", linewidth = 0.4) +
  scale_fill_gradient2(
    low = "#4575B4",
    mid = "white",
    high = "#D73027",
    midpoint = 0,
    name = "Standardized\nresponse"
  ) +
  labs(x = NULL, y = NULL) +
  theme_sci() +
  theme(
    axis.text.x = element_text(size = 7, color = "black", angle = 45, hjust = 1),
    axis.text.y = element_text(size = 8, color = "black"),
```

```
    axis.line = element_blank(),
    axis.ticks = element_blank(),
    legend.position = "right"
  )

fig7
save_sci(fig7, "Fig7_standardized_response_heatmap.tiff", height_mm = 90, width_mm = 180)

#### Exploratory PLS-PM model ####
if (!require(plspm)) {
  install.packages("plspm")
}
library(plspm)
library(tibble)
library(dplyr)

plspm_df <- main_df %>%
  dplyr::filter(Treatment %in% c("P2", "P3", "P4", "P5")) %>%
  dplyr::mutate(
    PAM_app = ifelse(PAM == "Yes", 1, 0),
    Bio_app = ifelse(Microbial inoculant == "Yes", 1, 0)
  ) %>%
  dplyr::select(
    PAM_app,
    Bio_app,
    SWC, WSA,
    AK, MBC,
    Root_biomass, Root_surface_area,
    Biomass, K_uptake
  ) %>%
  dplyr::mutate(
    dplyr::across(dplyr::everything(), as.numeric)
  ) %>%
  stats::na.omit()

print(dim(plspm_df))
print(head(plspm_df))

latent_vars <- c(
  "PAM",
  "Microbial inoculant",
  "Water_structure",
```

```
  "Fertility",
  "Root",
  "Growth_K"
)

path_matrix <- matrix(
  0,
  nrow = length(latent_vars),
  ncol = length(latent_vars)
)

rownames(path_matrix) <- colnames(path_matrix) <- latent_vars
path_matrix["Water_structure", "PAM"] <- 1
path_matrix["Fertility", "Microbial inoculant"] <- 1
path_matrix["Root", "Water_structure"] <- 1
path_matrix["Root", "Fertility"] <- 1
path_matrix["Growth_K", "Root"] <- 1

print(path_matrix)

blocks <- list(
  PAM = c("PAM_app"),
  Microbial inoculant = c("Bio_app"),
  Water_structure = c("SWC", "WSA"),
  Fertility = c("AK", "MBC"),
  Root = c("Root_biomass", "Root_surface_area"),
  Growth_K = c("Biomass", "K_uptake")
)

modes <- rep("A", length(blocks))

plspm_res <- plspm(
  Data = plspm_df,
  path_matrix = path_matrix,
  blocks = blocks,
  modes = modes,
  scaled = TRUE,
  boot.val = TRUE,
  br = 500
)

summary(plspm_res)
```

```
path_coefs <- plspm_res$path_coefs
outer_model <- plspm_res$outer_model
effects <- plspm_res$effects
boot_paths <- plspm_res$boot$paths

print("Path coefficients:")
print(path_coefs)

print("Outer model:")
print(outer_model)

print("Effects:")
print(effects)

print("Bootstrap paths:")
print(boot_paths)

if ("inner_summary" %in% names(plspm_res)) {
  print("Inner summary:")
  print(plspm_res$inner_summary)
}

print("GoF:")
print(plspm_res$gof)

lv_cor <- cor(plspm_res$scores, use = "pairwise.complete.obs")

ave_vec <- plspm_res$inner_summary$AVE
sqrt_ave <- sqrt(ave_vec)

fornell_larcker <- lv_cor
diag(fornell_larcker) <- sqrt_ave[colnames(fornell_larcker)]

print("Fornell-Larcker criterion:")
print(round(fornell_larcker, 3))
path_ci_table <- plspm_res$boot$paths %>%
  as.data.frame() %>%
  tibble::rownames_to_column("Path") %>%
  dplyr::rename(
    Beta = Original,
    Boot_mean = Mean.Boot,
```

```
    Boot_SE = Std.Error,
    CI_2.5 = perc.025,
    CI_97.5 = perc.975
  ) %>%
  dplyr::mutate(
    Significance = dplyr::case_when(
      CI_2.5 > 0 | CI_97.5 < 0 ~ "Significant",
      TRUE ~ "Not significant"
    )
  )

print("Path coefficients with bootstrap CI:")
print(path_ci_table)

r2_table <- plspm_res$inner_summary %>%
  as.data.frame() %>%
  tibble::rownames_to_column("Latent_variable") %>%
  dplyr::select(
    Latent_variable,
    Type,
    R2,
    AVE,
    Block_Communality,
    Mean_Redundancy
  )

print("R2 and model quality:")
print(r2_table)

reliability_table <- plspm_res$unidim %>%
  as.data.frame() %>%
  tibble::rownames_to_column("Latent_variable") %>%
  dplyr::rename(
    Cronbach_alpha = C.alpha,
    Composite_reliability = DG.rho,
    Eigenvalue_1st = eig.1st,
    Eigenvalue_2nd = eig.2nd
  )

print("Reliability and convergent validity:")
print(reliability_table)
write.csv(
```

```
  fornell_larcker,
  "result_tables/PLSPM_simplified_Fornell_Larcker.csv",
  row.names = TRUE
)

write.csv(
  path_ci_table,
  "result_tables/PLSPM_simplified_path_CI_table.csv",
  row.names = FALSE
)

write.csv(
  r2_table,
  "result_tables/PLSPM_simplified_R2_AVE_redundancy.csv",
  row.names = FALSE
)

write.csv(
  reliability_table,
  "result_tables/PLSPM_simplified_reliability_validity.csv",
  row.names = FALSE
)

calc_f2_safe <- function(target_lv, predictor_lv) {

  path_matrix_reduced <- path_matrix
  path_matrix_reduced[target_lv, predictor_lv] <- 0

  reduced_res <- plspm(
    Data = plspm_df,
    path_matrix = path_matrix_reduced,
    blocks = blocks,
    modes = modes,
    scaled = TRUE,
    boot.val = FALSE
  )

  r2_included <- plspm_res$inner_summary[target_lv, "R2"]
  r2_excluded <- reduced_res$inner_summary[target_lv, "R2"]

  f2 <- (r2_included - r2_excluded) / (1 - r2_included)
```

```
  data.frame(
    Path = paste(predictor_lv, "->", target_lv),
    Target = target_lv,
    Predictor = predictor_lv,
    R2_included = r2_included,
    R2_excluded = r2_excluded,
    f2 = f2
  )
}

f2_table <- dplyr::bind_rows(
  calc_f2_safe("Root", "Water_structure"),
  calc_f2_safe("Root", "Fertility")
) %>%
  dplyr::mutate(
    Effect_size = dplyr::case_when(
      f2 >= 0.35 ~ "Large",
      f2 >= 0.15 ~ "Medium",
      f2 >= 0.02 ~ "Small",
      TRUE ~ "Negligible"
    )
  )

print("f2 effect size for predictors of Root:")
print(f2_table)

write.csv(
  f2_table,
  "result_tables/PLSPM_simplified_f2_effect_size_Root.csv",
  row.names = FALSE
)
if (!dir.exists("result_tables")) {
  dir.create("result_tables")
}

write.csv(
  plspm_res$path_coefs,
  "result_tables/PLSPM_simplified_path_coefficients.csv",
  row.names = TRUE
)

write.csv(
```

```
  plspm_res$outer_model,
  "result_tables/PLSPM_simplified_outer_model_loadings.csv",
  row.names = FALSE
)

write.csv(
  plspm_res$unidim,
  "result_tables/PLSPM_simplified_unidimensionality.csv",
  row.names = TRUE
)

write.csv(
  plspm_res$effects,
  "result_tables/PLSPM_simplified_effects.csv",
  row.names = FALSE
)

write.csv(
  plspm_res$scores,
  "result_tables/PLSPM_simplified_latent_variable_scores.csv",
  row.names = FALSE
)

write.csv(
  plspm_res$boot$paths,
  "result_tables/PLSPM_simplified_bootstrap_paths.csv",
  row.names = TRUE
)

write.csv(
  data.frame(GoF = plspm_res$gof),
  "result_tables/PLSPM_simplified_GoF.csv",
  row.names = FALSE
)

if ("inner_summary" %in% names(plspm_res)) {
  write.csv(
    plspm_res$inner_summary,
    "result_tables/PLSPM_simplified_inner_summary_R2.csv",
    row.names = TRUE
  )
}
```

```
plot(plspm_res)
```
